# Enhanced insulin‐regulated phagocytic activities support extreme health span and longevity in multiple populations

**DOI:** 10.1111/acel.13810

**Published:** 2023-03-08

**Authors:** Deng Wu, Xiaoman Bi, Peihu Li, Dahua Xu, Jianmin Qiu, Kongning Li, Shaojiang Zheng, Kim Hei‐Man Chow

**Affiliations:** ^1^ School of Life Sciences, Faculty of Science The Chinese University of Hong Kong Hong Kong; ^2^ Key Laboratory of Tropical Translational Medicine of Ministry of Education, College of Biomedical Information and Engineering Hainan Medical University Haikou China; ^3^ Key Laboratory of Tropical Cardiovascular Diseases Research of Hainan Province Tumor Institute of The First Affiliated Hospital, Hainan Women and Children Medical Center, Hainan Medical University Haikou China

**Keywords:** extended longevity, healthy lifespan, innate immune system, insulin sensitivity

## Abstract

The immune system plays a central role in many processes of age‐related disorders and it remains unclear if the innate immune system may play roles in shaping extreme longevity. By an integrated analysis with multiple bulk and single cell transcriptomic, so as DNA methylomic datasets of white blood cells, a previously unappreciated yet commonly activated status of the innate monocyte phagocytic activities is identified. Detailed analyses revealed that the life cycle of these monocytes is enhanced and primed to a M2‐like macrophage phenotype. Functional characterization unexpectedly revealed an insulin‐driven immunometabolic network which supports multiple aspects of phagocytosis. Such reprogramming is associated to a skewed trend of DNA demethylation at the promoter regions of multiple phagocytic genes, so as a direct transcriptional effect induced by nuclear‐localized insulin receptor. Together, these highlighted that preservation of insulin sensitivity is a key to healthy lifespan and extended longevity, via boosting the function of innate immune system in advanced ages.

## Graphical abstract

The immune system plays a central role in aging. The findings illustrated that the innate immune system plays critical roles in shaping extreme health span and longevity. Monocytes from long‐lived individuals reveal enhanced life‐cycles and a M2‐like phenotype, which are associated with an insulin‐driven immune‐metabolic network that supports multiple aspects of such transformation. These highlighted that preservation of insulin sensitivity is a key to healthy lifespan and extended longevity.

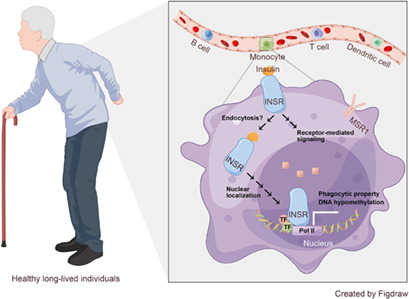

## 1 INTRODUCTION

The rapidly aging population around the world, particularly in developed countries, has urged policy makers to switch the focus of healthcare from delivering adequate treatments to small populations of patients with acute episodes of single diseases to providing sustainable measures for maintaining good health and avoid the development of multiple chronic age‐related conditions in the general population (Beard et al., 2016). The immune system plays a central role in many processes of age‐related non‐communicable diseases such as cardiovascular diseases, type 2 diabetes, and dementia (Rea et al., 2018). Activated immune functions, which frequently describe as inflammation, has been recognized as part of their pathophysiologies (Rea et al., 2018). However, accumulating evidence challenges this assumption and suggests that the immune system may instead get mounting adaptive responses to chronic stressors, prolonging the chances of survival of an organism (Rubinow & Rubinow, 2017; Soo et al., 2022). To address this argument, one possible way is to investigate the immune signatures in long‐lived individuals (LLIs; mean age >95 year old) and centenarians, the “aging champions” who achieved successful human aging and exhibited medical histories with remarkably low incidences of common age‐related disorders (Borras et al., 2016). Since inflammaging and immunosenescence is a common feature of chronological aging in ordinary people contributing to enhances risks of mortality at advanced age (Borras et al., 2016); this proposes that a better functioning immune system, with stronger pro‐survival and stress handling abilities, are likely at play in shaping extreme longevity.

The immune system can be schematically seen as two divisions. The ancestral/innate arm is mainly represented by monocytes, natural killer (NK) and dendritic cells (DC); whereas the adaptive arm is represented by the B and T lymphocytes. As if a functioning immune system requires a homeostatic balance between the two, gene expression profiles of circulating immune cells would likely reveal important clues that are crucial for achieving healthy aging. A recent single‐cell transcriptomic study reported that expansion of cytotoxic CD4 T cells is a unique immune signature among supercentenarians (Hashimoto et al., 2019); whereas previous bulk transcriptome studies proposed that shift in lymphocyte to myeloid cell ratio (Karagiannis et al., 2022), enhanced autophagy‐lysosomal function (Xiao et al., 2018); reduction in ribosomal biosynthesis (Xiao et al., 2022), or upregulated apoptotic Bcl‐xL (Borras et al., 2016) is however crucial to successful aging. It remains unclear if any common immune features unique to extreme longevity exist among LLIs regardless to their origins; and whether the associated molecular signatures can provide insights for practical translations.

By harnessing the wealth of single‐cell and bulk transcriptome datasets available in the public repositories (Table 1); we uncovered that significant induction of innate immune monocytes with enhanced lysosomal and phagocytic activity is a previously unrecognized, common, and unique immune signature among LLIs from various geographical origins and ethnicities. The life cycle of these monocytes in LLIs is enhanced and primed to a M2‐like macrophage phenotype. Monocytes are the major immune cells that express insulin receptor (INSR). Functional characterization revealed an insulin‐signaling centric immunometabolism network which supports multiple aspects of phagocytosis. Such reprogramming is associated to a skewed trend of DNA demethylation, particularly at the promoter regions of multiple phagocytic genes, so as a direct transcriptional effect induced by the nuclear INSR. Together, these findings highlighted that preservation of insulin sensitivity hence an active innate monocyte‐driven phagocytic activity is a defense mechanism in safeguarding healthy lifespan and extended longevity.

**TABLE 1:** Overview of datasets utilized in the study.

Transcriptome (Extreme longevity)			
Discovery cohort			
Dataset overview	Statistics	Long‐lived individuals (LLIs)	Immediate descendant's spouses (F1SP)
Location: Chengmai (ChM) County, Hainan Province, China	Sample no. (Total)	76	41
Accession no: HRA000656 (BIG Data Center, Beijing Institute of Genomics)	Female sample no.	58	40
Data type: Bulk RNA‐sequencing, white blood cell	Male sample no.	18	1
Reference citation: PMID 30352807	Age (Mean years)	102.2	59.9
Location: Lingao (LG) County, Hainan Province, China	Sample no. (Female only)	105	31
Location: Lingshui (LS) County, Hainan Province, China	Sample no. (Female only)	80	55
Accession no: CRA000515 (BIG Data Center, Beijing Institute of Genomics)	Age (LG+LS) (Mean years)	98.9	57.4
Data type: Bulk RNA‐sequencing, white blood cell			
Reference citation: PMID 35476452			
Validation cohort #1			
Dataset overview	Statistics	Long‐lived sibling	Control
Location: Leiden City, South Holland Province, Netherlands	Sample no. (Total)	50	50
Accession no: GSE16717 (Gene Expression Omnibus, NCBI)	Female sample No.	24	26
Data type: Expression microarray, peripheral blood	Male sample No.	26	24
Reference citation: PMID 22247756	Female age (Mean years)	94.31	59.04
	Male age (Mean years)	92.56	65.1
Validation cohort #2			
Dataset overview	Statistics	Supercentenarians	Control
Location: Japan (Predicted to be nation‐wide)	Sample no. (Total)	7	5
Accession No: SC2018 (Riken, http://gerg.gsc.riken.jp/SC2018/)	Female sample no.	5	3
Data type: Single cell RNA‐sequencing, peripheral blood	Male sample no.	2	2
Reference citation: PMID 31719197	Female age (Mean years)	110	66.7
	Male age (Mean years)	110	65

Transcriptome (Normal aging)		
GTEx cohort		
Dataset overview	Statistics	Normal aging
Location: Unspecified	Sample no. (Total)	755
Accession No: https://gtexportal.org/home/datasets (Gtex database)	Female sample no.	254
Data type: Bulk RNA‐sequencing, whole blood cell	Male sample no.	501
Reference citation: PMID 23715323	Age (Mean years)	20–79

Transcriptome (Healthy control samples for scRNA)		
COVID‐19 Cell atlas cohort		
Dataset overview	Statistics	Healthy individuals
Location: Unspecified	Sample no. (Total)	8
Accession no: https://www.covid19cellatlas.org/index.patient.html (Covid19 cell atlas)	Female sample no.	4
Data type: Single cell RNA‐sequencing, peripheral whole blood	Male sample no.	4
Reference citation: PMID: 32514174	Age (Mean years)	Unspecified

Transcriptome (Diseases association)				
Alzheimer's disease blood cohort				
Dataset overview	Statistics	Alzheimer's disease (AD)	Mild cognitive impairment (MCI)	Non‐demented control
Location: Not provided	Sample no. (Total)	204	134	249
Accession no: GSE140829 (Gene Expression Omnibus, NCBI)	Female sample no.	104	62	139
Data type: Expression microarray, peripheral blood	Male sample no.	100	72	110
Reference citation: https://www.biorxiv.org/content/10.1101/2019.12.13.875112v1 (Pre‐print)	Female age (Mean years)	73.05	71.89	73.359
	Male age (Mean years)	72.94	74.44	74.045
Type 2 diabetes blood cohort				
Dataset overview	Statistics	Type 2 diabetes (T2DM)	Non‐diabetic control	
Location: Not provided	Sample no. (Total)	7	6	
Accession no: GSE156993 (Gene Expression Omnibus, NCBI)	Female sample no.	3	4	
Data type: Expression microarray, peripheral blood mononuclear cells	Male sample no.	4	2	
Reference citation: Not provided from the Gene Expression Omnibus, NCBI	Female age (Mean years)	51	42.5	
	Male age (Mean years)	55.25	38.5	
Coronary artery disease blood cohort				
Dataset overview	Statistics	Coronary Artery Disease (CAD)	Control	
Location: Not provided	Sample no. (Total)	49	48	
Accession no: GSE180081 (Gene Expression Omnibus, NCBI)	Female sample no.	21	26	
Data type: Expression microarray, peripheral blood mononuclear cells	Male sample no.	27	22	
Reference citation: PMID: 34479557	Female age (Mean years)	64.29	57.69	
	Male age (Mean years)	61.11	57.23	
Alzheimer's disease brain cohort				
Dataset overview	Statistics	Alzheimer's disease (AD)	Non‐demented control	
Location: Religious Orders Study and Memory and Aging Project (ROSMAP)	Sample no. (Total)	24	24	
Accession No: syn18485175 (AD Knowledge Portal)	Female sample no.	12	12	
Data type: Single‐nucleus RNA‐sequencing, brain cortex samples (Brodmann Area 10)	Male sample no.	12	12	
Reference citation: PMID: 31042697	Female age (Mean years)	85.33	87.42	
	Male age (Mean years)	86.08	83.75	

Epigenome (Extreme longevity)			
Europe blood cohort			
Dataset overview	Statistics	Nonagenarians	Middle‐age controls
Location: Uncertain but likely from Europe due to author affiliation, samples are of Caucasian ethnicity	Sample no. (Total)	19	19
Accession no: GSE33233, GSE30870 (Gene Expression Omnibus, NCBI)	Female sample no.	Unspecified	19
Data type: Whole‐genome bisulfite sequencing	Male sample no.	Unspecified	0
Reference citation: PMID 26161907	Female age (Mean years)	Sex unspecified (92.63)	Unspecified
	Male age (Mean years)		N/A

Insulin receptor promotor occupancy + Transcriptome			
Insulin receptor promotor occupancy			
Dataset overview	Statistics	Non‐starved (Insulin 10 nM)	Starved (Insulin 0 nM)
Accession no: GSE107335 (Gene Expression Omnibus, NCBI)	Sample no. (Total)	6	6
Data type: ChIP‐sequencing, HepG2	IR antibody	2	2
Reference citation: PMID 30955890	RNA pol antibody	2	2
	Input	2	2
Insulin‐induced expression profiling			
Dataset overview	Statistics	Non‐starved (Insulin 10 nM)	Starved (Insulin 0 nM)
Accession no: GSE107336 (Gene Expression Omnibus, NCBI)	Sample no. (Total)	9	9
Data type: Bulk RNA‐sequencing, HepG2			
Reference citation: PMID 30955890			

## 2 RESULTS

### 2.1 A consensually activated innate immune signature in healthy LLIs

**FIGURE 1:**
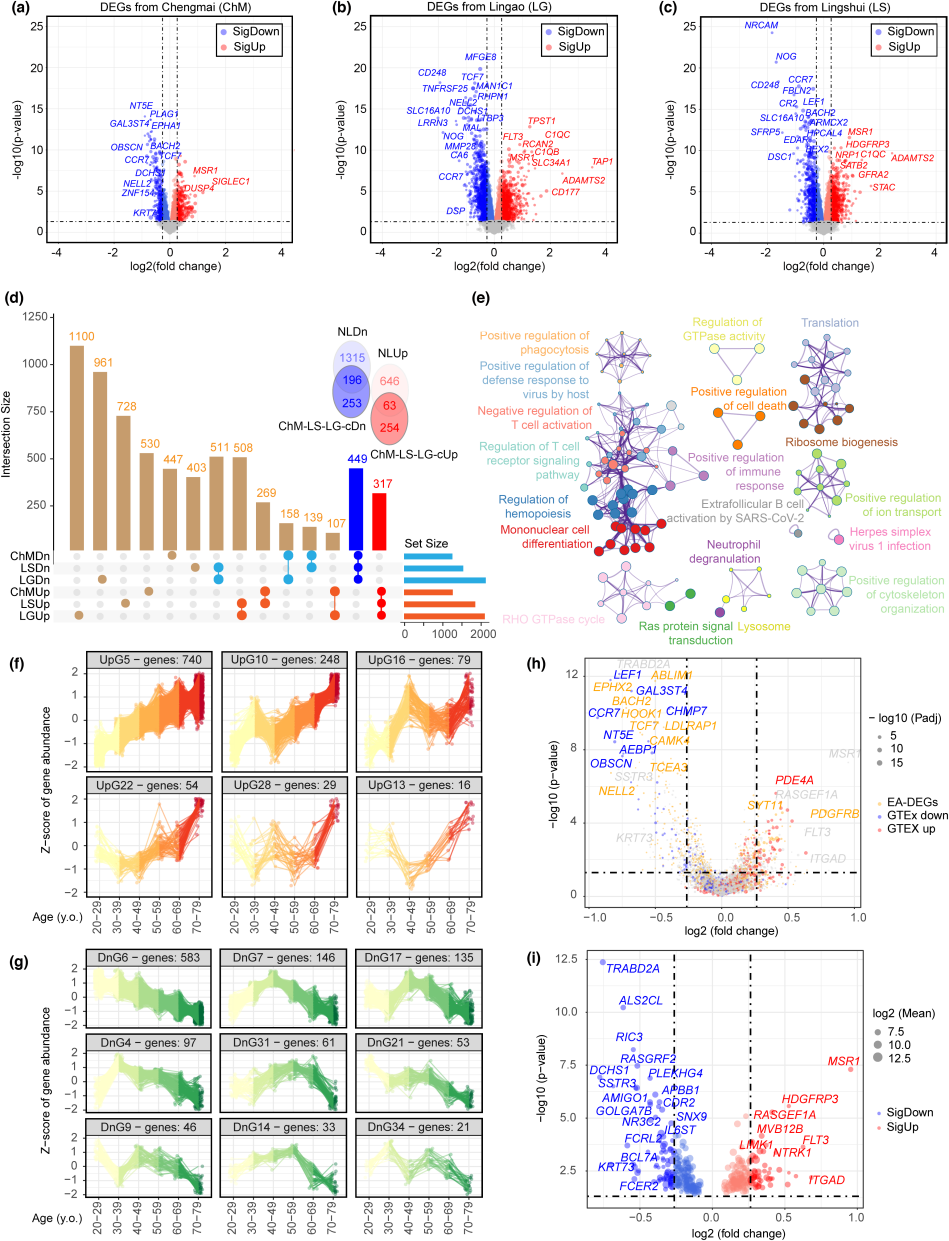
Conserved longevity and healthy aging (LHA) genes identification across multiple long‐lived populations. (a–c) Volcano plots of the differentially expressed genes (DEGs) between long‐lived individuals (LLIs) and their children's spouse (F1SP) from Chengmai (a), Lingao (b), and Lingshui (c). Genes with p‐values smaller than 0.05 and fold changes >1.2 were regarded as significantly changed (Red: upregulated; Blue: downregulated). (d) Upset plot of the shared genes across the entire Hainan cohort. The commonly up‐and down‐regulated genes were marked as ChM‐LS‐LG‐cUp and ChM‐LS‐LG‐cDn, respectively. These were compared to those identified from the Netherlands' Leiden (NL) Longevity Study (NLUp: upregulated; NLDn: downregulated). (e) Over‐representation analysis (ORA) of common LHA genes identified from (d) using the Metascape platform. Every single node represented an enriched term and two nodes were linked if their Kappa similarities were higher than 0.3. Similar functional terms were clustered together and were displayed using the same color. Node size was proportional to the number of enriched genes. (f–g) Expression pattern plots of the common aging‐associated genes in the human whole blood transcriptomes obtained from GTEx database. The upregulated and downregulated common aging‐associated genes were manually extracted, if the trend of changes persist uniformly >50 years old (y.o.). (h) Volcano plot of the shared genes across the Hainan cohort. Commonly found age‐associated genes identified from the GTEx were colored by red and blue corresponded to the up‐ and down‐regulation during normal aging. Yellow dots points represented the common aging associated‐genes identified from the 14,983 individuals of European ancestry (EA) studies. (i) Volcano plot of the LHA genes after eliminating the common aging‐associated genes showed in (H).

It has been reported that aspects of the aging process could be inferred from the gene expression profiles of peripheral blood cells. To investigate if common health‐related signature exists among LLIs or centenarians of different geographical origins; initial analyses of three independent sets of white blood cells transcriptomic data, obtained from a total of 261 disease‐free, healthy LLIs and 127 young controls (i.e., immediate descendant's spouses, F1SP) originated from Cheng Mai (ChM) (Xiao et al., 2018), Lingshui (LS) (Xiao et al., 2022), Lingao (LG) (Xiao et al., 2022) Counties of the Hainan province were performed (Table S1). With the geographical‐specific sets of differentially expressed genes (DEGs) (Figure 1a–c); commonly dysregulated DEGs (i.e., cDEGs), including 317 up‐ and 449 downregulated genes, were identified (Table S2, nominal *p* < 0.05). Indeed all these commonly deregulated genes were also identified by an alternative way of analysis if samples from all these geographical sites were first integrated together via batch effect correction (Table S2, adjusted *p* < 0.01). This therefore validated the robustness of these commonly deregulated genes. The non‐randomness of the cDEGs was confirmed by a premutation test via a random selection and overlapping estimation of the same number of genes as found in each set of DEGs. Such calculation revealed that a significantly lower number of genes (i.e., only 27 up‐ and 22 down‐regulated) were expected to be common (Figure S1A). Further comparison was made with the list of DEGs (i.e., 709 up‐ and 1511 down‐regulated) obtained from the nonagenarians in the Netherlands' Leiden (NL) Longevity Study (Passtoors et al., 2012). Around 19.87% of up‐ and a higher 43.65% of down‐regulated genes from the Hainan cDEGs were indeed common to those from the Netherland's dataset (Figure 1d), suggesting a certain degree of similarities in blood profiles exist among these individuals of different ethnicity. Among the commonly upregulated were those that promote M2 polarization [e.g., Macrophage scavenger receptor‐1 (*MSR1*) (Chen et al., 2022), ATP‐binding cassette subfamily G member‐1 (*ABCG1*) (Sag et al., 2015), sphingomyelin synthase‐2 (*SGMS2*) (Deng et al., 2021)], insulin signaling [e.g., insulin receptor (*INSR*)] and associated lipid biosynthesis metabolism [e.g., steroid sulfatase (*STS*) (Giovagnoni et al., 2021), Serine palmitoyltransferase (*SPTLC2*) (Rotthier et al., 2010)]; whereas those commonly downregulated were those that promote apoptosis [e.g., caspase‐6 (*CASP6*) (Cowling & Downward, 2002), TNF receptor superfamily member 10a (*TNFRSF10A*; *aka death receptor 4*, *DR4*) (Micheau, 2018)] (Table S3). Next, with a focus on our Hainan discovery cohorts, subsequent gene functional analysis using the Metascape tool revealed that the over‐represented biological pathways identified from each independent Hainan DEGs set were also very similar to one another (Figure S1B, Table S4). Common upregulated pathways concurrently supported the innate immune responses, such as monocyte differentiation, activation of lysosomes, metal ion transport, and cytoskeleton remodeling for phagocytosis. In the contrary, activities related to the adaptive immune response mediated by the T‐ and B‐cells became less robust (Figure 1e, Figure S1C,D).

As these non‐diseased, healthy LLIs or nonagenarians have lived their lives to the extreme of human longevity, it is expected that their immune system are also subjected to changes related to the normal aging process, in addition to the changes underlying extreme longevity. To differentiate between the two, a larger scale analysis was performed with whole blood transcriptome profiles of 755 healthy subjects aged between age 20 and 79 years obtained from the genotype‐tissue expression (GTEx) database (Figure 1f,g, Figure S1E). With a total of 4148 DEGs categorized into 36 groups based on their expression patterns along the age scale (Figure S1E), 14 groups were selected due to their consensual up‐ or down‐ward trends observed at age 50 years and beyond, plus one group (Group 16) that revealed strong and robust gene upregulation at age 70–79 (Figure 1f,g). These genes, together (Up: 1166; Down: 1175), were then defined as the “GTEx‐common age‐associated genes” (Table S5). For any genes occurred on this list, along with the 1497 common age‐associated genes identified from a meta‐analysis of six European ancestry (EA) studies (38.2–78.2 year old) (Peters et al., 2015), were filtered out from cDEGs list curated from the Hainan cohort (Figure 1h), leaving a net of 159 up‐ and 211 down‐regulated genes that truly reflected changes associated with extreme longevity (Figure 1i, Table S6). Subsequent gene functional characterization by the Metascape tool of this extreme longevity and healthy aging gene list (LHA) identified an insulin signaling‐centric immunometabolism network (Figure S2A). At the metabolic side, insulin signaling facilitates the biosynthesis of sphingolipid (Bryan et al., 2015), amino and nucleotide sugars (Tafesse et al., 2015) that known to facilitate the recognition of foreign particles by macrophage and neutrophils. At the immune side, the signaling also activates a set of downstream pathways that are known to facilitate phagocytosis, including plasma membrane organization, vesicle‐mediated plasma membrane transport, Wnt signaling and tyrosine kinase signaling network (Figure S2B,C, Table S7).

Mounting evidence had indicated that the immune system adapts and reprograms upon chronic stress via initiating an allostasis response (Rubinow & Rubinow, 2017) that allows physiological recalibration and creates conceptual spaces for adaptive responses that may result in new biological set points in order to support survival (Rubinow & Rubinow, 2017). Such remodeling is frequently found in non‐communicable, age‐related chronic conditions, including the early mild cognitive decline (MCI) and advanced Alzheimer's disease (AD), type 2 diabetes (T2DM), and coronary artery disease (CAD). Surprisingly, as compared to their respective age‐matched controls, peripheral blood mononuclear cells (PBMCs) of individuals of T2DM (41.16–53.42 year old); CAD (57.48–62.50 year old); MCI (73.37–73.51 year old) or AD (72.92–73.51 year old) consensually revealed a transcriptome signature resembling to that observed among LLIs (Figure S3A–D). Further tracing analysis performed between the samples from the MCI and AD groups which respectively represented the early and late stage of Alzheimer's‐related dementia revealed that changes occurred to the curated list LHA genes indeed matched better to the advanced disease stage (Figure S3C,D). These patterns suggested that despite being non‐diseased and healthy, the peripheral blood profiles of LLIs were pre‐set to an activated status resembling to those who had been adapted to tolerate chronic stresses.

### 2.2 Enhanced lysosomal‐phagocytic function in professional phagocytes is associated with healthy lifespans and extreme longevity

**FIGURE 2:**
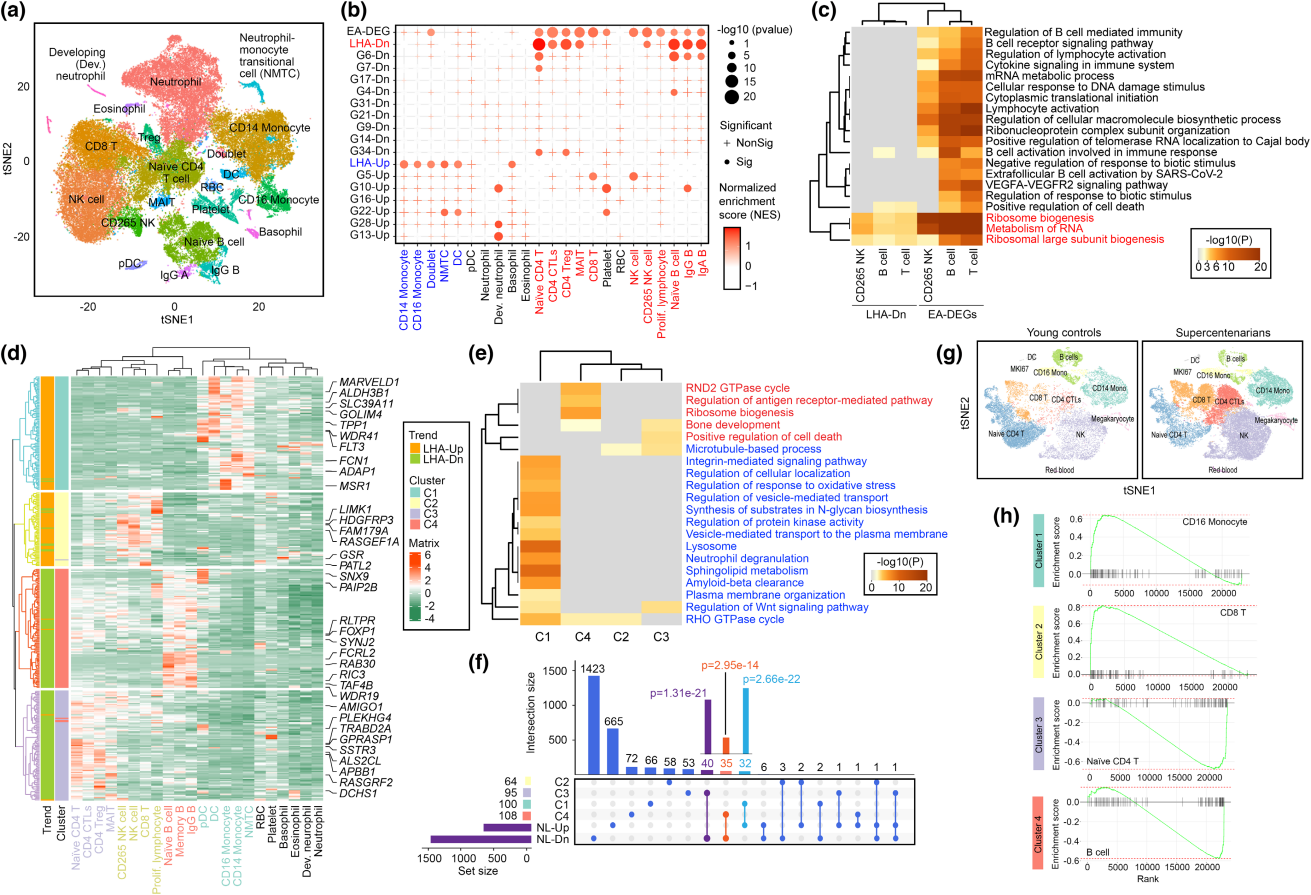
Robust activation of professional phagocytes in LLIs. (a) TSNE plot of whole blood single cell transcriptome profiles. Various cell types were differentially colored. DC: dendritic cells, NK: nature killer, RBC: red blood cell, Tregs: regulatory T cells. (b) Enrichment of LHA and common aging‐associated genes across various immune cell types. The enrichment significance was assessed using GSEA. Point size represented the *p*‐value. Positive normalized enrichment score (NES) (Red points) represents the gene list that highly expressed in the corresponding cell type. Terms were considered as significant when the corresponding *p*‐values were smaller than 0.05. These were indicated as a dot, otherwise that would be labelled with a plus symbol. (c) Comparison of the ORA pathway enrichment with LHA downregulated genes versus the common aging‐associated genes in lymphocytes. (d), Cell type‐specific expression analysis of LHA genes. Unsupervised clustering was used to group LHA genes into four clusters. (e) ORA enrichment results of the LHA genes and they could be grouped as four clusters. (f) Upset plot illustrated the similarities of LHA genes implicated in the four clusters with the DEGs identified from the Netherlands (NL). Significance was evaluated via the hypergeometric test. (g) TSNE plot of single cell transcriptome profiles of PBMCs (provided by Hashimoto et al) between young adults and supercentenarians. (h) GSEA plots of the gene list from the four clusters. Comparisons were made between young adults and supercentenarians in various immune cell types.

The blood contains many subtypes of white blood cells and they could be differentially regulated in the context of aging. To delineate the specific cell‐type contribution in the common immune signatures identified among LLIs and Netherland nonagenarians, blood cell type‐specific molecular characters were profiled with reference to single‐cell transcriptomes (Wilk et al., 2021). Among a total of 61,880 while blood cells explored (Figure 2a), 22 major cell types were identified based on their established markers (Figure S4) (Wilk et al., 2021). From there, pre‐defined gene lists, such as the LHA genes, DEGs list curated from the European ancestry (EA) studies (EA‐DEG), and genes clustered in selected groups (Figure 1f,g) were mapped to different cells types (Table S8). As in details, list of downregulated LHA genes and those broadly defining the common age effects (Figure 1f,g) were found to be enriched predominantly in the adaptive immune lymphocyte lineage, covering the majority of the T‐ [i.e., Naïve CD4T, Regulatory T cells (CD4 Treg), CD4+ T cells with cytotoxic activity (CD4 CTLs), Mucosal associated invariant T cells (MAIT), CD8+ T,] and B‐ (i.e., Naïve B, IgG+ B, Memory B) cells; and to a lesser extent the natural killer (NK) effective lymphocytes (i.e., CD265+ NK) of the innate immune system (Figure 2b, Table S8). These downregulated LHA genes were characterized as a set of bona fide pathways needed for lymphocyte activation, cytokine signaling, as well as those involved in RNA metabolism and ribosome biogenesis (Figure 2c). When comparison on these pathways was made between the two groups of individuals of different lifespans, this revealed that the adaptive immune system of LLIs were better preserved and be less phenotypically altered than those of normally aged individuals (Figure 2c). In LLIs, only significant suppression of ribosomal biogenesis was observed among these lymphocytic lineages (Figure 1e and 2c). Ribosomal biosynthesis is an energy‐intense process, consuming >60% of cellular energy (Zhou et al., 2015), these activities are highly activated among the lymphocytes (Figure S5). In LLIs, a reduced ribosomal activity may facilitate the conservation of cellular energy for other purposes during the process of aging (Xiao et al., 2022). In contrast, the upregulated LHA genes were highly expressed in the innate immune cells, particularly the monocytes [i.e., CD14 Mono, CD16 Mono], monocyte transitional cells (MTC) [i.e., Neutrophil‐to‐monoyte transitional cell] and dendritic cells (DC) (Figure 2b, Table S8).

To further characterize the differentially expressed gene signature patterns in these cells, each gene was assigned with a score based on its relative expression in every single cell, followed by unsupervised clustering of these genes into different gene clusters (Figure 2d, Table S9). Four major clusters were substantially identified. Majority of the upregulated LHA genes enriched in classic professional phagocytes, including the monocytes and DCs (Cluster 1), so as the lymphocytic NK cells and CD8‐positiive cytotoxic T cells (CD8 T) (Cluster 2) whose major protective mechanism is also related to phagocytosis. For the downregulated LHA genes, these were over‐represented in both T (Cluster 3) and B cells (Cluster 4) (Figure 2d). Gene functional characterization of upregulated LHA genes in Cluster 1 using the Metascape tool revealed their predominant roles in mediating phagocytosis at both the cell membrane (i.e., plasma membrane organization, vesicle‐mediated transport, sphingolipid metabolism, integrin‐mediated signaling) and intracellular levels (i.e., N‐glycan biosynthesis, lysosome, RHO GTPase cycle) (Figure 2e, Table S10).

In contrast to Cluster 1, Cluster 2 is a small cluster, the related genes were more associated with RHO GTPase signaling and microtubule‐based process for phagocytic engulfment enriched in NK cells and cytotoxic CD8+ T cells (Figure 2e, Table S10). For the rest were the downregulated LHA genes that potentially facilitate ribosome biosynthesis and antigen‐receptor mediated pathway in B cells (Cluster 4); so as those promoting the turnover of T cells (Cluster 3) (Figure 2e, Table S10). To validate such activated professional phagocytic signature as well as the more bioenergetic‐efficient lymphocytes network are the immune features associated with extreme longevity, DEG set from the Netherlands' Leiden (NL) Longevity Study (Passtoors et al., 2012) was compared, which consistently revealed similar patterns of changes for genes in Clusters 1, 3, and 4 (Figure 2f). From there, 32% of Cluster 1 genes, 35% of Cluster 2, and 40% of Cluster 4 were consistently matched (Figure 2f). On the other hand, cellular specific changes were further validated by comparing to the single‐cell transcriptomic dataset of supercentenarians from Japan (Hashimoto et al., 2019) (Figure 2g, Figure S6). Consistently, for the upregulated LHA genes in Cluster 1, they were uniquely enriched in CD16‐positive monocytes. Those in Cluster 2 were highly expressed in the CD8 T cells. Among downregulated LHA genes, genes in Cluster 3 were enriched in the naïve CD4‐positive T (Naïve CD4 T) and those in Cluster 4 were found predominantly in B cells (Figure 2h). These cross‐validation analyses using samples of different ethnicities together supported the robust gain‐of‐phagocytic function among the innate professional phagocytes is a previously unrecognized and common immune signature unique to LLIs of multiple ethnicities.

### 2.3 Activated phagocytic monocytes prime a M2‐like alternatively activated macrophage signature in LLIs

**FIGURE 3:**
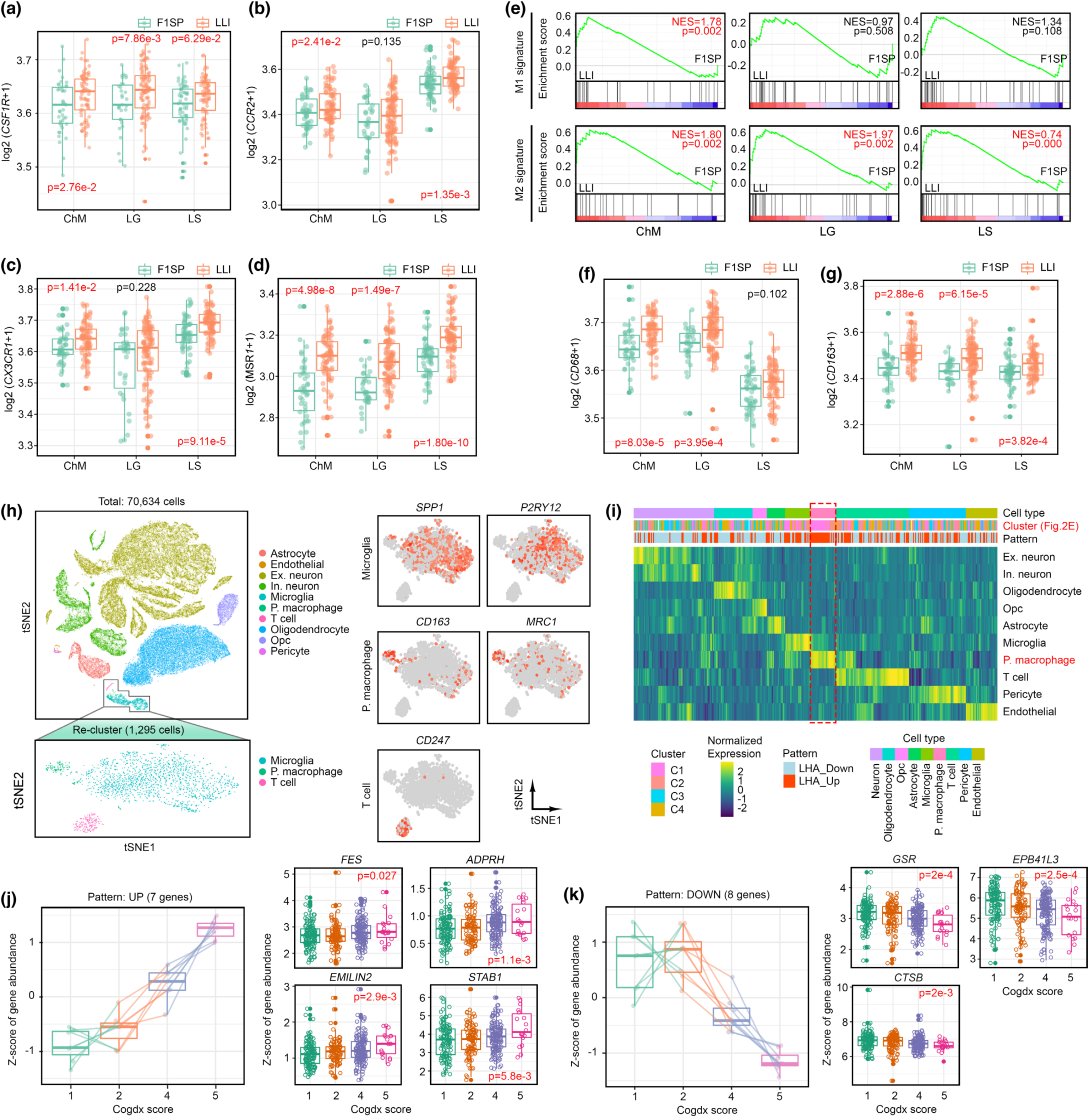
Activated phagocytic monocytes prime a M2‐like alternatively activated macrophage signature in Hainan LLIs. (a–d) Tukey boxplots (showing the median line, interquartile range (IQR) boxes, and 1.5 × IQR whiskers) for the expression levels of (a) *CSF1R*, (b) *CCR2*, (c) *CX3CR1*, and (d) *MSR1* in the Hainan cohort. Data were presented as log‐transformed expression values, adjusted for sex and library type. (e) M1‐like macrophage signatures were not significantly altered between LLIs and F1SPs (upper panel), but M2‐like signatures were increased in LLIs (bottom panel). (f–g) Tukey boxplots (showing the median line, interquartile range (IQR) boxes, and 1.5 × IQR whiskers) for the expression levels of (f) *CD68* and (g) *CD163* in the Hainan cohort. (h) TSNE plot of 70,635 cells from the brain prefrontal cortex samples of Mathys et al. (Mathys et al., 2019) dataset. Immune cell cluster was further isolated and re‐clustered using a higher resolution to dissociate the T cells, peripheral macrophage (P.macrophage), and brain resident‐microglia with their respective markers. Ex: excitatory neurons; In: inhibitory neurons; Opc: Oligodendrocyte progenitor cells. (i) Evaluation of the relative expression levels of the LHA genes across the distinct cell types in the human brain. The average gene expression of the LHA genes between cell types was compared and were further clustered to identify the peripheral macrophage‐specific expressed LHA genes, as highlighted by the dash box. (j) Tukey boxplots for the peripheral macrophage associated‐LHA genes found being (j) up‐ and (k) down‐regulated during the course of cognitive impairment. Values represent median z‐score of gene expression. Statistical significance was calculated by one‐way ANOVA.

Apart from the above qualitative changes, the relative quantities of these immune cell populations were estimated by the CIBERSORTx deconvolution calculation using their respective cell type‐specific markers (Figure S7). Among all the detectable cell types, monocytes ratio was significantly increased among all three groups of Hainan LLIs as compared to their F1SP. In contrary, the reverse trend was found in the Naïve CD4 T cells (Figure S7A). Indeed, similar downward trend of the latter was observed as well during the normal aging process (Figure S7B). This inferred that the increase in monocyte cellular ratio, but not the changes in naïve CD4 T cells, was the only quantitative change closely associated to extreme longevity. To further investigate the details of such changes, markers related to events occur during the monocyte life cycle were analyzed. Referencing previous studies which reported that colony‐stimulating factor 1 receptor (*CSF1R*) that promotes proliferation of monocytes, macrophages, and dendritic cells was significantly downregulated during the course of ordinary aging (Duong et al., 2022; Hearps et al., 2012), here this gene was however found to be upregulated among LLIs as compared to their F1SP (Figure 3a, Figure S8A). Subsequently, both C‐C chemokine receptor type 2 (*CCR2*) and its receptor C‐X‐C motif chemokine receptor‐1 (*CXCR1*)—crucial factors that mediate the recruitment of monocytes to the peripheral tissues—were also upregulated (Figure 3b,c) and such changes was again unique to extreme longevity (Figure S8B–E). Upon reaching various tissues, circulating monocytes differentiate, and polarize into tissue‐infiltrating macrophages. Previous studies reported that the process of monocyte‐to‐macrophages differentiation is only associated with ~2% of global genes expression adjustment (Martinez et al., 2006). Based on this, the transcriptomic signatures of peripheral monocytes shall be useful to interpret the M1/M2‐like phenotypes of the macrophages derived from them. Among the significantly altered LHA genes, the M2‐like transcription factor macrophage scavenger receptor‐1 (*MSR1*) was significantly upregulated in both Hainan LLI (Figure 3d) and Leiden nonagenarian cohorts (Figure S8F). Globally, the M2‐like but not M1‐like signatures were found as well (Figure 3e; Figure S8G), which was further confirmed by the elevated expression of the classic M2‐like markers: macrosialin (*CD68*) and *CD163* (Figure 3f,g).

As our earlier analysis revealed that the peripheral white blood cell transcriptome profiles of LLI somehow resembles to the profiles emerged under the reprogramming stress of chronic diseases (Figure S3), this suggested that similar changes could occur at macrophage level (Figure S9). Using the AD model, re‐analysis of a set of brain tissue single‐nucleus transcriptomic data harvested from 48 age‐matched samples (Control: 24; AD: 24) (Mathys et al., 2019) indeed supported this prediction (Figure 3h). Further analysis indicated that infiltrated peripheral macrophages, as compared to other resident immune cells (i.e., microglia and T cells) in the brain, revealed enriched expression of 22 LHA genes upregulated in LLIs (Figure 3i, Figure S9). Despite so, these genes, however, had their expression manifested differently during disease progression (Figure 3j,k, Figure S10). For instance, tyrosine kinase *FES* and stabilin‐1 (*STAB1*) that activate phagocytosis (Park et al., 2009; van der Wel et al., 2020), ADP‐ribosylarginine hydrolase *ADPRH* that inhibits interferon‐gamma signaling (Menzel et al., 2021) for activating phagocytosis, and the less functionally characterized extracellular glycoprotein elastin microfibril interfacer 2 (*EMILIN2*) were significantly upregulated during the progression of cognitive decline (Figure 3j). In contrast, genes significantly downregulated along disease progression were ones more functionally diverse. These included the glutathione‐disulfide reductase (*GSR*) in regulating redox homeostasis; erythrocyte membrane protein band 4.1‐like three (*EPB41L3*) that enable cytoskeletal protein‐membrane anchor activity, and cathepsin b (*CTSB*) required for macrophage inflammasome activation (Chevriaux et al., 2020) (Figure 3k). Collectively, these suggested that increased phagocytosis capacity—an M2‐like signature—is generally observed in brain infiltrated peripheral macrophages in advanced disease; and that resembles the prediction from the circulating monocytes of the LLIs (Figure S3). Together, these alternatively confirmed that the innate immune system of the disease‐free individuals who lived to extreme longevity is pre‐activated and reprogrammed to withstand chronic stresses.

### 2.4 Enhanced insulin sensitivity and oxidative phosphorylation is associated with the functional reprogramming of the monocytes

**FIGURE 4:**
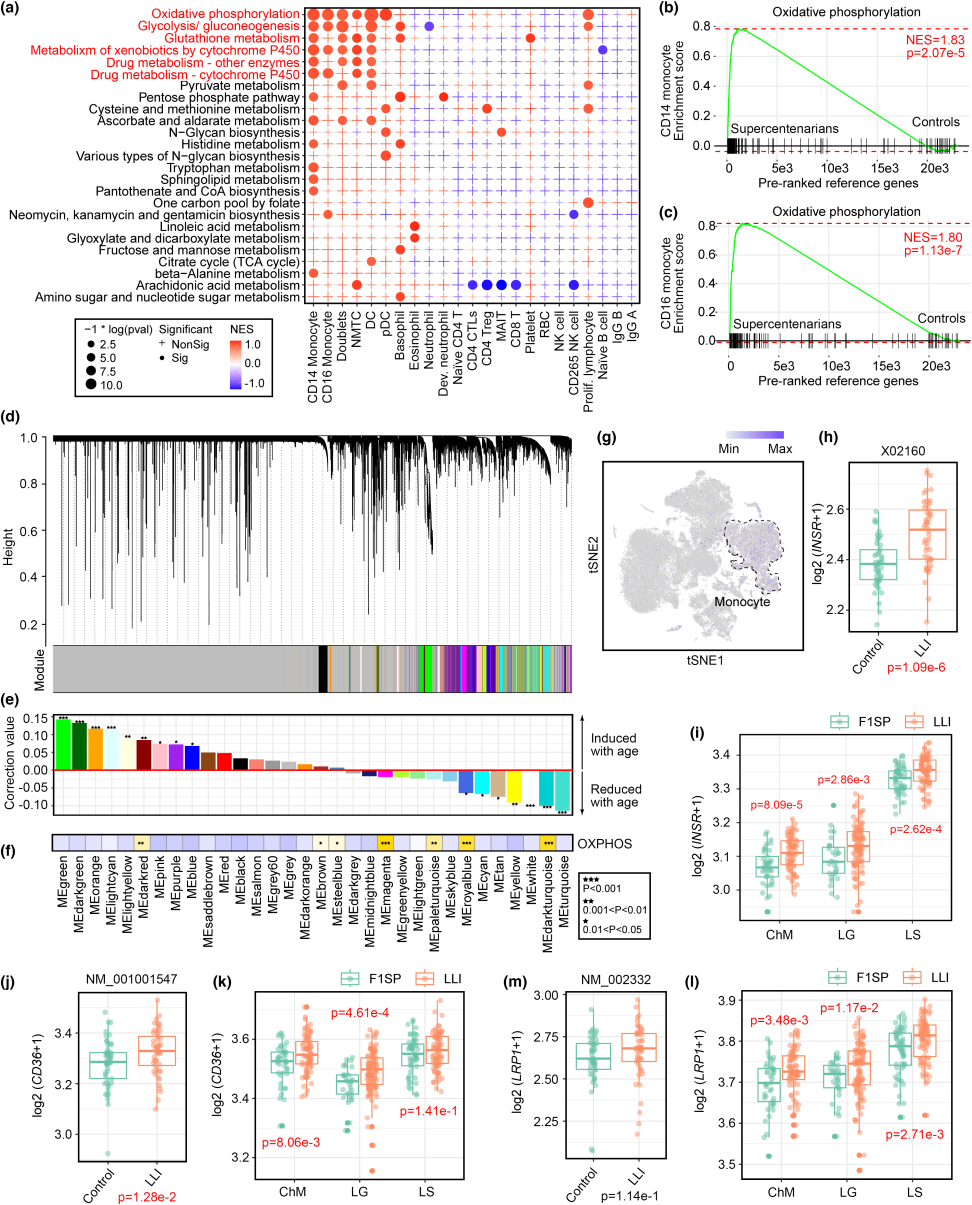
Enhanced insulin sensitivity and oxidative phosphorylation is associated with the functional reprogramming of the monocytes. (a) Dot plot revealing the relative expression level of genes involved in KEGG metabolic pathways in various immune cells (as identified in Figure 2a). Significance was evaluated using GSEA. Red and blue dots respectively represented a high or low normalized enrichment score (NES). Point size corresponded to the *p*‐value. Significance was considered when *p*‐value was smaller than 0.05, and would be indicated as a dot, or else as a plus symbol. (b–c) Genes involved in oxidative phosphorylation were enriched in both CD14 and CD16 monocytes harvested from Japanese supercentenarians as compared to the corresponding controls. (d) WGCNA dendrogram presented the co‐expressed gene modules in CD14 monocytes. (e) Age‐related modules were identified by the Pearson correlation coefficient test, the relationship between aging and the first principle component from each module was evaluated. (f) Enrichment analysis of oxidative phosphorylation (OXPHOS) genes in various modules. Significance was evaluated by the hypergeometric test. (g) TSNE plot showing the *INSR* expression was uniquely found in monocytes. (h–k) Tukey boxplots showing (h–i) *INSR*, (j–k) *CD36* and (M‐L) *LRP1* gene expression in subjects from either Leiden or Hainan cohort. Significance was evaluated by Limma (for Leiden) and DESeq2 (Hainan), respectively.

The study of energy expenditure has deep roots in understanding aging and lifespan in all species. Reduced energy production from metabolic active tissues and organ is one of the prominent drivers of aging. Therefore, energy conservation via streamlining the limited resources to sustain essential functions had been developed, for instance a decline in cellular ribosomal biogenesis during the post‐developmental phase is a mechanism for promoting healthy aging and longevity (Xiao et al., 2022). Indeed, such energy‐saving changes were also observed during the course of aging (Figure 2). Immune defense is energy demanding, competitions for limited nutrients, fuel and energy occurs between the acquired and innate immune systems. The reduced energy demand among lymphocytes observed proposed that a more favorable global metabolic environment to support the gain in surveilling phagocytic functions among professional phagocytes in LLIs. To better understand the role of fuel metabolism in these cells, their intrinsic metabolic signatures were profiled. With 1711 non‐redundant genes belonging to 87 metabolic pathways extracted from the Kyoto Encyclopedia of Genes and Genomes (KEGG) database, their relative expression levels among different types of immune cells were compared in non‐diseased samples of different datasets (Hashimoto et al., 2019; Wilk et al., 2021). Among all possible immune blood cells detected, the innate immune professional phagocyte lineages, particularly the CD14‐ and CD16‐positive monocytes and DCs, were ones consistently revealed strong activities in oxidative phosphorylation (OXPHOS) (*P*_CD14_ = 0.001, *NES*_CD14_ = 1.340; *P*_CD16_ = 0.001, *NES*_CD16_ = 1.304), glutathione metabolism (*P*_CD14_ = 0.018, *NES*_CD14_ = 1.291; *P*_CD16_ = 0.151, *NES*_CD16_ = 1.171) and those associated to cytochrome P450 (*P*_CD14_ = 0.005, *NES*_CD14_ = 1.327; *P*_CD16_ = 0.009, *NES*_CD16_ = 1.282) (Figure 4a, Figure S11), highlighting their intrinsic demand in metabolic requirements (Table S11). Such observation was in huge contrast to the adaptive immune cell lineages, where activities of these pathways were barely at minimal and not enriched. Instead, anaerobic pathways such as the pentose phosphate pathway (*P*_basophil_ = 0.010, *NES*_basophil_ = 1.466; *P*_developing neurotrophil_ = 0.045, *NES*_developing neurotrophil_ = 1.504); glycolysis/gluconeogenesis (*P*_basophil_ = 0.027, *NES*_basophil_ = 1.393; *P*_doublets_ = 0.001, *NES*_doublets_ = 1.298; *P*_proliferating lymphocyte_ = 0.041, *NES P*_proliferating lymphocyte_ = 1.254); and fructose and mannose metabolism (*P*_basophil_ = 0.027, *NES*_basophil_ = 1.393) dominated in these cells (Table S11). With an emphasis on the energy‐generating OXPHOS pathway, these genes were indeed also highly expressed in monocytes of supercentenarians from Japan (*P*_CD14_ = 2.07 e‐5, *NES*_CD14_ = 1.83, *P*_CD16_ = 1.13 e‐7, *NES*_CD16_ = 1.80) (Figure 4b,c, Table S11), suggesting that is a cellular‐specific feature in promoting extreme longevity. To further validate so, transcriptome profiles of monocytes harvested from 1183 individuals of common lifespans ranging from 44 to 83 years old were analyzed (Reynolds et al., 2014). By the weighted gene co‐expression network analysis (WGCNA), a total of 36 modules characterized based various gene expression pattern identified (Figure 4d). Within which 16 modules were significantly correlated to chronological aging (Figure 4e). Genes involved in OXPHOS were indeed clustered in modules whose expression trends were negatively correlated to age in individuals of ordinary life spans (Figure 4f). This validated that the enhanced OXPHOS is a unique feature in monocytes related to extreme longevity.

As hinted from our early functional analysis, the LHA gene set characterizing the signatures both the overall peripheral blood profiles of LLIs indeed highlighted an insulin signaling immunometabolism network (Figure S2). Insulin signaling has multifaceted stimulatory effects on OXPHOS (Nisr & Affourtit, 2014). This is also realized in insulin resistance—a condition where insulin signaling is compromised—can lead to the opposite effects. Indeed, *INSR* was uniquely expressed in monocytes (Figure 4g), and its expression was significantly upregulated among both Hainan LLIs (Figure 4h) and Netherland nonagenarians (Figure 4i). This, with concurrent induction of other cell surface proteins, such as the *MSR1* (Figure 1i and 3d); cluster of differentiation 36 (*CD36*) (Figure 4j,k) and LDL receptor‐related protein‐1 (*LRP1*) (Figure 4l,m), promotes insulin signaling and cellular insulin sensitivity via facilitating INSR trafficking, recycling and its downstream immunometabolism effect in these cells (Actis Dato & Chiabrando, 2018; Cavallari et al., 2018; Puchalowicz & Rac, 2020). Together, these data suggested that the enhanced phagocytic signatures of the innate immune monocytes and the descendent M2‐like macrophages was likely supported by a robust insulin signaling and OXPHOS network, for fulfilling the elevated energy demand from active phagocytosis.

### 2.5 LLI phagocytic genes are targets of insulin receptor and insulin‐regulated transcription factors

**FIGURE 5:**
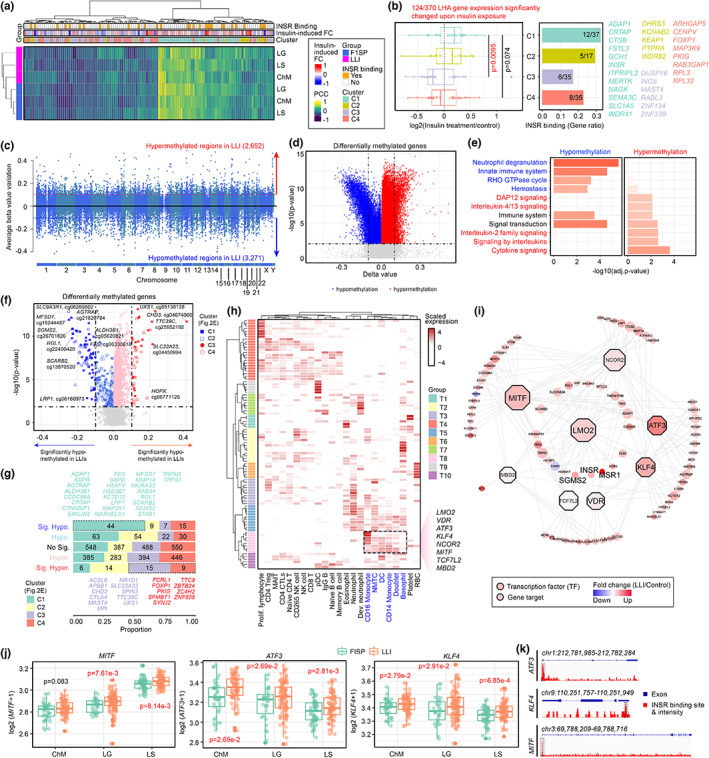
LLI phagocytic genes are targets of insulin receptor and insulin‐regulated transcription factors. (a) Heatmap summarizing the Pearson correlation coefficient (PCC) relationship between INSR and LHA genes under the influence of insulin stimulus. (b) Tukey boxplot illustrating the expression trends of insulin‐responsive LHA genes after insulin stimulus. Bar plot on the right representing the ratio of insulin‐sensitive LHA genes with INSR binding sites to the total number of genes in each clusters (Defined in Figure 2e). (c) Comparison of global methylation profiles from nonagenarians and middle‐aged individuals (Heyn et al., 2012). (d) Volcano plot of the global methylation state between nonagerians and middle‐aged controls. Entries with absolute delta beta value greater than 0.1 and adjusted *p*‐value smaller than 0.01 were considered as significant. (e) Functional enrichment of the genes adjacent to hypo‐ and hyper‐methylated regions. Genes in proximal to methylated regions were extracted from the HumanMethylation450 annotation file, enrichR web server was used to analyze the hypo and hyper‐methylated associated genes. (f) Volcano plot of the differentially methylated regions across the DNA sequence of LHA genes. Differentially methylated regions (DMR) with absolute beta value variation larger than 0.1 and the adjusted p‐value smaller than 0.01 were considered as significant. (g) Statistics of the proportion of cell type‐specific LHA genes in each methylation pattern. (h) Heatmap summarizing an array of transcription factors (TFs) at play in regulating the expression of differentially methylated LHA genes. Clustering of TFs was performed based on their expression levels in various immune cell types. (i) Network revealing the inter‐relationship between phagocyte‐specific transcription factors and their target LHA genes. (j) Tukey boxplots showing *MITF*, *ATF3*, and *KLF4* gene expression in subjects from the Hainan cohort. Significance was evaluated by DESeq2. (k) Genome browser shots of *MITF*, *ATF3*, and *KLF4* gene promoter regions near the transcription start site. Gene structure is shown in blue. INSR binding intensities at various locations were shown, as measured using the ChIP‐Seq dataset^5^. High‐confidence binding regions were highlighted in dash boxes.

Recent study revealed that activated INSR can directly associate with promoters and regulate target gene expression (Hancock et al., 2019). With the list of 3976 genes containing INSR‐binding sites at their promoters (Hancock et al., 2019), cross‐comparison to those LHA genes in the four clusters revealed that expression of those belonged to Clusters 1 and 2 predominantly correlated positively to insulin‐stimulation; whereas those in Clusters 3 and 4 were reversely correlated (Figure 5a,b). Additionally, higher ratio of the LHA genes in Clusters 1 and 2 were confirmed to be directly regulated by INSR, as inferred by the presence of INSR binding sites at their core promoter regions (Figure 5b). For the LHA genes that were unlikely INSR gene targets, we reasoned that their changes could be a result of alternative downstream effects of insulin signaling, including effects at the epigenetic level (Ling, 2020). Previously, the Monozygotic Twin Study which analyzed blood samples from twins ranged from 48–61 years old had revealed that global DNA methylation of white blood cell is associated with insulin resistance (Zhao et al., 2012). Here, if the robust and sensitive insulin signaling is one of the keys in favoring extreme longevity, it is possible that the unique transcriptomic changes observed were related to changes in DNA methylation status. By comparing the DNA methylomes between nonagenarians (Mean age = 92.2 year old) and middle‐aged individuals (Mean age = 59.8 year old) (Heyn et al., 2012), delta beta values obtained from the unsupervised analysis indicated a strong negative skewness, inferring a more hypomethylation trend among centenarians (Figure 5c,d). With a total of 2652 and 3217 significantly hyper‐ and hypo‐methylated regions mapped correspondingly to 1606 and 1817 genes (Table S12), their functional features were subsequently analyzed on the Enrichr platform based on the Reactome 2022 pathway (Table S13). Enhanced Rho GTPase signaling (Junttila, 2018) and neutrophil degranulation (Gierlikowska et al., 2021) supporting the phagocytic activities of innate immune system were identified from the significantly hypomethylated genes (Figure 5e, Table S13). Concurrently, an interleukin (Junttila, 2018; Ross & Cantrell, 2018) and DAP12 cytokine signaling network (Tomasello & Vivier, 2005) needed to facilitate the maturation of lymphocyte‐lineage of the adaptive immune system was likely deactivated, hinted by the corresponding gene hypermethylation status (Figure 5e, Table S13). These methylome‐based changes therefore alternatively supported findings from the transcriptomic analyses performed in other independent datasets.

With the same approach, a more refined analysis was performed on the genomic regions located at proximity to the curated list of LHA genes, which revealed a total of 119 differentially methylated regions (DMR) (Figure 5f). From there, a total of 44 and 75 hyper‐ and hypo‐methylated regions respectively corresponded to 36 and 49 LHA genes identified (Figure 5g,h). Among the significantly hypermethylated LHA genes, 24/36 of them were those clustered to C3‐4, representing the suppressed adaptive immune functions in LLIs (Figure 2d,e, 5g). In the contrary, for the significantly hypomethylated genes, they belonged mainly to C1, the predominant cluster representing induced monocytic activities in LLIs. Indeed, 26/49 of them also belonged to the LHA gene list (Figure 2d,e, 5g), and most of them were non‐redundant to the direct insulin targets (Figure 5b). Changes in DNA methylation alters the local chromosome structure, hence the promoter accessibility to various regulatory factors. By a gene promotor analysis, 97 transcription factors regulating the upregulated phagocytic LHA genes were identified (Figure 5h). From there, eight of them were uniquely expressed among the innate professional phagocytes (Figure 5h, dash box), functioning as an interlinked regulatory network (Figure 5i). While most of them revealed upregulated trends in Hainan LLIs, expression levels the microphthalmia‐associated transcription factor (*MITF*) which robustly regulate genes in the innate immune signaling pathways (Yu et al., 2021); the Kruppel‐like factor‐4 (*KLF4*) that governs monocyte differentiation (Feinberg et al., 2007); and the activating transcription factor‐3 (*ATF3*) that promotes macrophage migration and M2 polarization via suppressing the M1‐like fate (Sha et al., 2017) were only ones significantly induced (Figure 5j, Figure S12). Notably, these transcription factors were also direct targets of nuclear INSR, where robust binding sites were identified at their core promoter regions (Figure 5k). Together, both changes observed in the global DNA methylome, and transcription factor abundance support potential influence of insulin, explaining how it may regulate the innate immune system of LLIs.

## 3 DISCUSSION

Our study constitutes a holistic analysis, illustrating the changes in immune functional network and molecular signatures associated with extreme health span and longevity common to multiple groups of “aging champions” across continents. By performing an integrated analysis using multiple blood transcriptome and DNA methylome data from subjects of different geographical and ethnicity backgrounds; this has led to an unexpected finding on the insulin‐sensitive, activated innate immune monocytes, with enhanced lysosomal and phagocytic activities. The findings on the activation of the innate immune monocytes have not been reported before, and such changes are found to be supported by an insulin signaling‐driven immunometabolic network that remained sensitive and active among these subjects. The robust insulin action is tailored to the INSR‐presenting monocytes, supporting their intrinsic metabolic demands crucial for further differentiation and polarization as M2‐like macrophages with robust lysosomal and phagocytic activities.

The relationship between immunity and longevity, particularly extreme longevity, has been established (Sansoni et al., 2008). Here we revealed that the peripheral blood immune signatures of disease‐free healthy individuals who lived to extreme longevity were indeed resembling to those who were at much younger age but suffering from various chronic conditions. This observation was in fact counter‐intuitive to the main stream assumption that an altered immune signature is part of the pathoetiologies. Indeed, accumulating evidence emerged recently challenges this perspective, rather it is proposed that the immune signatures seen could be a reflection of a continual adaptive responses and physiologic recalibration to chronic stressors, leading to new biological set points that favors organismal survival under stressful conditions (Rubinow & Rubinow, 2017). In lower organisms like *Caenorhabditis elegans*, a recent study indicated that enhanced stress resistance among long‐lived mutants is a key in determining longevity (Soo et al., 2022). Here, our findings also re‐emphasize the fact that inflammation as part of the normal immune defenses has been selected through the evolution, its critical role in supporting survival of an organism shall not be overlooked. From this perspective, here we speculate that the common changes observed in individuals of extreme longevity are indeed pre‐set to withstand chronic stress, even before the emergence of chronic diseases. The new set points are associated with a better preservation of the declining adaptive immune system, in agreement with many previous finding (Karagiannis et al., 2022; Moskalev et al., 2020); so as an unexpected increment of the innate monocytes population, accompanied with activated phagocytic and lysosomal functions.

Immune defenses are energy demanding (McDade et al., 2016), and the efficiencies of energy metabolism and resources allocation are strongly related to the lifespan of an organism. From this perspective, the preference in boosting the innate immune function over that of the adaptive system proposes an underlying metabolic advantage during the extreme aging condition. Differential energy requirements between the two systems have been proposed (McDade et al., 2016). As compared to the opposite trends in the acquired immune system, the innate immune system imposes a lower upfront developmental cost, but higher operating costs when being activated. In disease‐free long‐living individuals whose body nutritional functions have also simultaneously suffered from an age‐related decline, this inevitable condition shall favor the energy investment toward the development of innate immune defenses as a barricade toward unknown encounters of pathogens and infections (McDade et al., 2016). Along the same vein, pathway analysis indeed also suggested a less active ribosomal biosynthesis network among the adaptive immune lineages. This could indeed be metabolically beneficial by sparing the limited fuel metabolites for the rest of the body immune function, as indeed even at resting conditions, nearly half of ribosomal RNA synthesized in the lymphocytes are degraded without even participating in any defense‐related protein synthesizing purposes (Cooper, 1970). Moreover, reduced protein synthesis/turnover in ribosomes is a conserved mechanism to alleviate cellular senescence stress in multiple cell types (Xiao et al., 2022). Functionally, monocytes, DCs and macrophages also play important upstream roles in regulating T cell fate as well, via presenting various antigens, secretion of cytokines, and other co‐stimulatory signals (Chu et al., 2020).

Phagocytosis is an integral part of the innate immune system. Immunologically‐silent phagocytosis of foreign pathogens and autologous apoptotic cells is crucial to maintaining tissue homeostasis, wound healing, and innate immune balance. While phagocyte dysfunction has been associated with tissue aging and potentially related to age‐related diseases; its direct linkage to lifespan and longevity remains elusive, except in one study reported in laboratory mice (Guayerbas & De La Fuente, 2003). Here we provide a direct evidence in human illustrating that enhanced monocyte phagocytic activities is the key immune signature that distinguishes disease‐free aging champions from those who lived to only normal lifespans. Moreover, our network‐based analysis revealed that an activated insulin signaling‐centric immunometabolic network is a potential driver underlying the enhanced lysosomal‐phagocytic activities in the insulin‐sensitive monocytes. Recently, it has been proposed that the innate immune cells are also capable in building immune memory characteristics. This “trained immunity” describes a persistent hyperresponsive phenotype of innate immune cells after brief stimulation (Tercan et al., 2021). In this essence, insulin signals could be a potential underrecognized stimulus, introducing memories in monocytes and macrophages. Such effect is likely maintained by distinct epigenetic and metabolic mechanism that persists for at least months, if not for years due to the associated cellular reprogramming effect.

The effect of insulin on epigenetics has been illustrated reversely by the case of insulin resistance, which is associated with global DNA hypermethylation (Zhao et al., 2012). The peripheral blood cell DNA methylome analysis indeed echoed with the transcriptomic finding, not only it revealed that the global DNA methylome tend to be more hypomethylated among the LLIs; but the hypomethylated regions were indeed mapped to genes that facilitate the activation of phagocytosis in monocytes, allowing easier access of phagocytosis‐regulating transcription factors like *MITF*, *ATF3*, and *KLF4*, which also happens to be gene targets of nuclear INSR. While classically, insulin signals are conducted via its receptor tyrosine kinase and a downstream signaling network initiated at the cell membrane level, it is recently revealed that insulin‐INSR complex can directly associates with target gene promoters via interactions with coregulators (Hancock et al., 2019). We noticed that a significant proportion of non‐differentially methylated genes in Clusters 1–4 were indeed direct targets of nuclear INSR, illustrating the multifaceted effects of insulin in monocytes. To what extent these different downstream effects of insulin signaling are involved in reprogramming the monocytes, so as the detailed mechanisms of it in regulating their DNA methylome remains unknown and these shall warrant future investigations. Nonetheless, our findings from the LLIs suggest that preservation of monocyte insulin sensitivity is a clue to healthy aging and extended longevity. While strategies leading to enhanced recycling and cell surface abundance of INSR specifically on monocytes could be one possible therapeutic approach; alternatively, maintenance of insulin sensitivity can easily be achieved and extended to whole body level via lowering circulating insulin levels. The latter could be practically achieved via lifestyle based adjustment, such as adhering to a tailored low‐starchy carbohydrate diet, intermittent fasting program, or an exercise strategy that are properly guided by physicians and healthcare professionals. The findings together explained how the whole‐body endocrine status regulates aging and longevity via modulating the immune functions.

## 4 METHODS AND MATERIALS

### 4.1 Data accession

*Datasets for identifying the extreme longevity and healthy aging gene list (LHA)—* Raw reads count matrix and sample information of long live individuals (LLIs) from Hainan province were requested from Xiao et, al. (Xiao et al., 2018, 2022); both of which were deposited in the Genome Sequence Archive (Wang et al., 2017) of the BIG Data Center with the following accession numbers: HRA000656 and CRA000515. Transcriptional profiles of nonagenarians in the Netherlands' Leiden (NL) Longevity Study were obtained from Gene Expression Omnibus (GEO) with accession number: GSE16717 (Passtoors et al., 2012).

*Datasets for defining the GTEx‐common age‐associated genes* —RNA‐seq raw counts matrix of the whole blood cells from the healthy person as well as their phenotypes were downloaded from the genotype‐tissue expression (GTEx) project (v8). About 1497 chronological age‐associated genes identified by individuals of European ancestry were obtained from the corresponding report (Peters et al., 2015).

*Datasets of peripheral blood gene expression profiles in age‐associated chronic disease* —Transcriptome profiles of PBMCs from individuals of mild cognitive impairment/ Alzheimer's disease (MCI/AD) (Nachun et al., 2019), type 2 diabetes mellitus (T2DM), and coronary artery disease (CAD) were downloaded from the GEO with accession numbers: GSE140829, GSE156993, and GSE180081, respectively.

*Single‐cell RNA‐seq datasets for the identifying cell type‐specific characteristic* — Single‐cell transcriptomics of whole blood cells were downloaded from COVID‐19 Cell Atlas (https://www.covid19cellatlas.org/) provided by Blish lab (Wilk et al., 2021). Single‐cell transcriptomic dataset of PBMCs between supercentenarians and control (Hashimoto et al., 2019) was downloaded from http://gerg.gsc.riken.jp/SC2018. Single‐cell transcriptome profiles of brain cells was obtained from the synapse database with accession number: syn18485175 (Mathys et al., 2019).

*Datasets used to predict the upstream insulin effect—* DNA methylomes were downloaded from GSE30870 and GSE33233 (Heyn et al., 2012; Simo‐Riudalbas et al., 2015). Chip‐seq of insulin receptor binding site across the genomic and RNA‐seq profiles of insulin treatment were downloaded from GEO database with GSE107335 and GSE107334 (Hancock et al., 2019), respectively.

### 4.2 Identification of differentially expressed genes (DEGs) in LLIs

Differential gene expression analyses between LLIs and F1SPs of all Hainan studies were performed with DESeq2 (v 1.30.1) using the default settings. Note that as different sample pre‐treatment methods, sequencing batches and library types existed among the datasets of ChM, LG, and LS; therefore, we performed differentially expressed genes (DEGs) separately first based on geographical location to avoid potential biases rooted from technologies and experimental treatments; followed by any posthoc comparisons performed. Therefore, factors related to library type, batch effect, and sex were included in the design formula in ChM dataset; whereas for the LG dataset, effect of library type was considered while the potential effect of normal aging was analyzed for removal. To cover a comprehensive set of longevity‐associated genes, nominal *p*‐value <0.05 were considered as differentially expressed. The overlap genes between the three populations were extracted and visualized by UpSetR package (v1.4.0). Differentially expressed transcripts between nonagerians and control from the nonagenarians in the Netherlands' Leiden (NL) Longevity Study were identified using limma (v3.46.0) with the linear model controlling for sex covariates. Transcripts with *p*‐value <0.01 were considered as significantly changed, which were further enrolled in validating the conservation of LHA genes across ethnicities.

### 4.3 Identification of common aging‐associated genes

Common age‐related DEGs of whole blood cells were identified corresponding transcriptome profiles obtained from GTEx using DESeq2 (v1.30.1) with the likelihood ratio test (LRT). Sex and Hardy scale were included in the LRT to remove their potential effect on aging. Age‐associated genes were identified at a significance threshold of adjusted *p* < 0.05. Significant age‐associated genes were scaled using the remove batch effect function in limma package by setting the sex and Hardy score as covariates. The scaled gene expression matrix was used as input for degPatterns functions in DEGreport (v1.26.0) to estimate the gene expression similarities based on their Pearson relationships. Gene groups were obtained by using the diana function in diana package. Genes included in the clusters with progressive and consistent trends in the aging period (>50‐year‐old) were manually selected as the common aging gene. As a result, 15 groups were selected.

To validate the robustness of the manual selection approach, we have also custom‐devised a statistical method/metric to identify the age‐associated pattern based on the mean value of the scale scores of each age group generated by degPatttern (Table S14). By calculating the slope value using the following formula:

$$\begin{array}{cc} \text{Slope value}\;=\; & \left(\text{Mean}\;{\text{Value}}_{60-69}-\text{Mean}\;{\text{Value}}_{50-59}\right)\\ & \times \left(\text{Mean}\;{\text{Value}}_{70-79}-\text{Mean}\;{\text{Value}}_{60-69}\right). \end{array}$$

For any slope value of each individual group that is greater than “0” means a consistent variation beyond 50 years old. Using this approach, 16 patterns were suggested to be selected (Table S14, orange highlights). This “calculated” group list was indeed in an almost perfect match (14/16 matched) with our “manually” selected list (i.e., Group name highlighted in red in Table S14 for easy comparison), except only three clusters, i.e., Groups 40, 32, and 16.

For Group 40, it was selected from the calculation, yet it was not included in our analysis as it contains only two genes, so that was omitted in subsequent analyses. For Group 32, since the mean Z‐score of gene abundance fell within similar range at 60–69 (−0.16494) and 70–79 (−0.20438), implying no obvious changes between the two age groups so that was omitted from the subsequent analysis as well. Our decision on selecting Group 16 was based on the fact that genes in it were highly and consistently induced in the age 70–79 period, as reflected by the mean Z‐score of gene abundance difference, which shall play significant roles in advanced ordinary aging processes (Figure 1f).

Consensus gene modules in CD14 monocytes across 1202 samples with age range from 44 to 83 years old were defined using the weighted gene co‐expression network analysis (WGCNA) (v1.71). The normalized probe expression matrix was downloaded from GEO using GEOquery (2.58.0) and was adjusted by the concentration value for each cell type using the remove batch effect function in limma (v3.46.0). Unsupervised clustering between samples identified 19 outliers and were subsequently removed. The blockwiseModules function was used to identify the co‐expressed modules with the following parameters: unsigned topological overlap matrix, minimum size of 30 probe for each module, dissimilarity threshold with 0.1, weighting parameter β was set as eight based on pickSoftThreshold function. The first principal component of each module was used to evaluate their relationship to age. TCA cycle and OXPHOS pathways associated genes were extracted from MSigDB (v7.4), and their enrichment significance were evaluated using hypergeometric test.

### 4.4 Deconvolution of immune cell types between LLIs and controls

To gain insight into the cellular composition variation in LLIs, CIBERSORTx web server (Newman et al., 2019) was used to estimate the immune‐cell subtype proportions by deconvolution of the bulk blood tissue. The signature matrix file LM22 was used as the reference to calculate the estimated cell type composition in each sample. The default setting was used in CIBERSORTx and the cell types with higher proportions of zero number (>50%) were filtered.

Further information on methods is available in the Supplemental Information.

## AUTHOR CONTRIBUTIONS

Kim Hei‐Man Chow, Shaojiang Zheng, and Kongning Li conceptualized and supervised the study. Deng Wu and Xiaoman Bi performed bioinformatics analysis and drafted the manuscript. Peihu Li, Dahua Xu, and Jianmin Qiu collected the raw data and optimized the analysis. Deng Wu and Kim Hei‐Man Chow revised and discussed the manuscript. All authors reviewed and gave final approval to the manuscript.

## CONFLICT OF INTEREST STATEMENT

The authors have no conflict of interest to declare.

## Supporting information


Figure S1.

Figure S2.

Figure S3.

Figure S4.

Figure S5.

Figure S6.

Figure S7.

Figure S8.

Figure S9.

Figure S10.

Figure S11.

Figure S12.

Table S1.

Table S2.

Table S3.

Table S4.

Table S5.

Table S6.

Table S7.

Table S8.

Table S9.

Table S10.

Table S11.

Table S12.

Table S13.

Table S14.

## Data Availability

All analysis have been carried using freely available software packages. Custom code used to analyse the RNA‐seq data and datasets generated and/or processed in the current study is available on https://github.com/KimChow‐Lab/LLIImmune/.
