# Supplementary figures and images for: Enhanced insulin‐regulated phagocytic activities support extreme health span and longevity in multiple populations
Source: Aging Cell. 2023 Mar 8;22(5):e13810. doi: 10.1111/acel.13810 (PMC10186610; doi:10.1111/acel.13810)

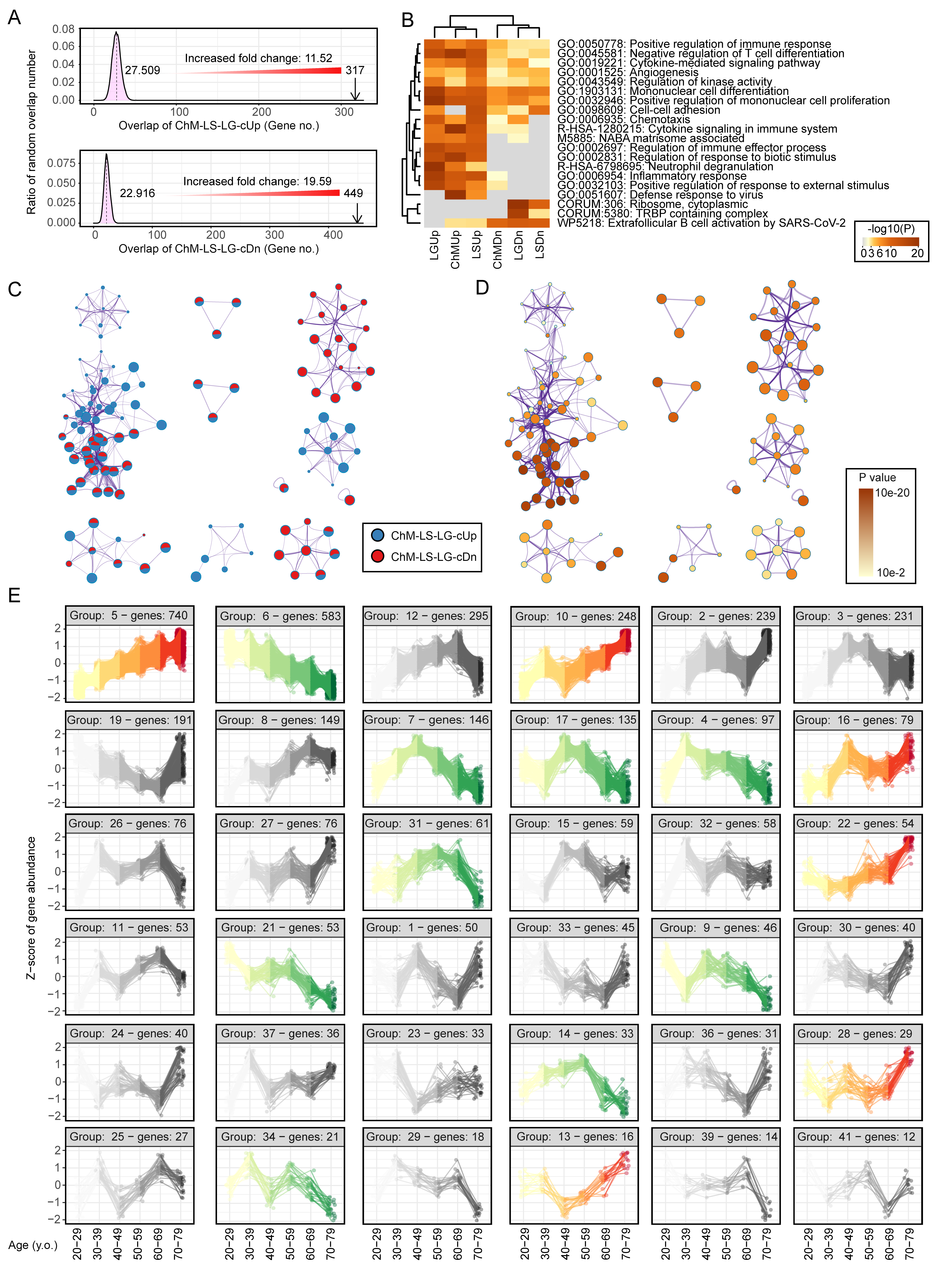

Supplement: Supplementary file 1 — Figure S1. [file ACEL-22-e13810-s008.tif]

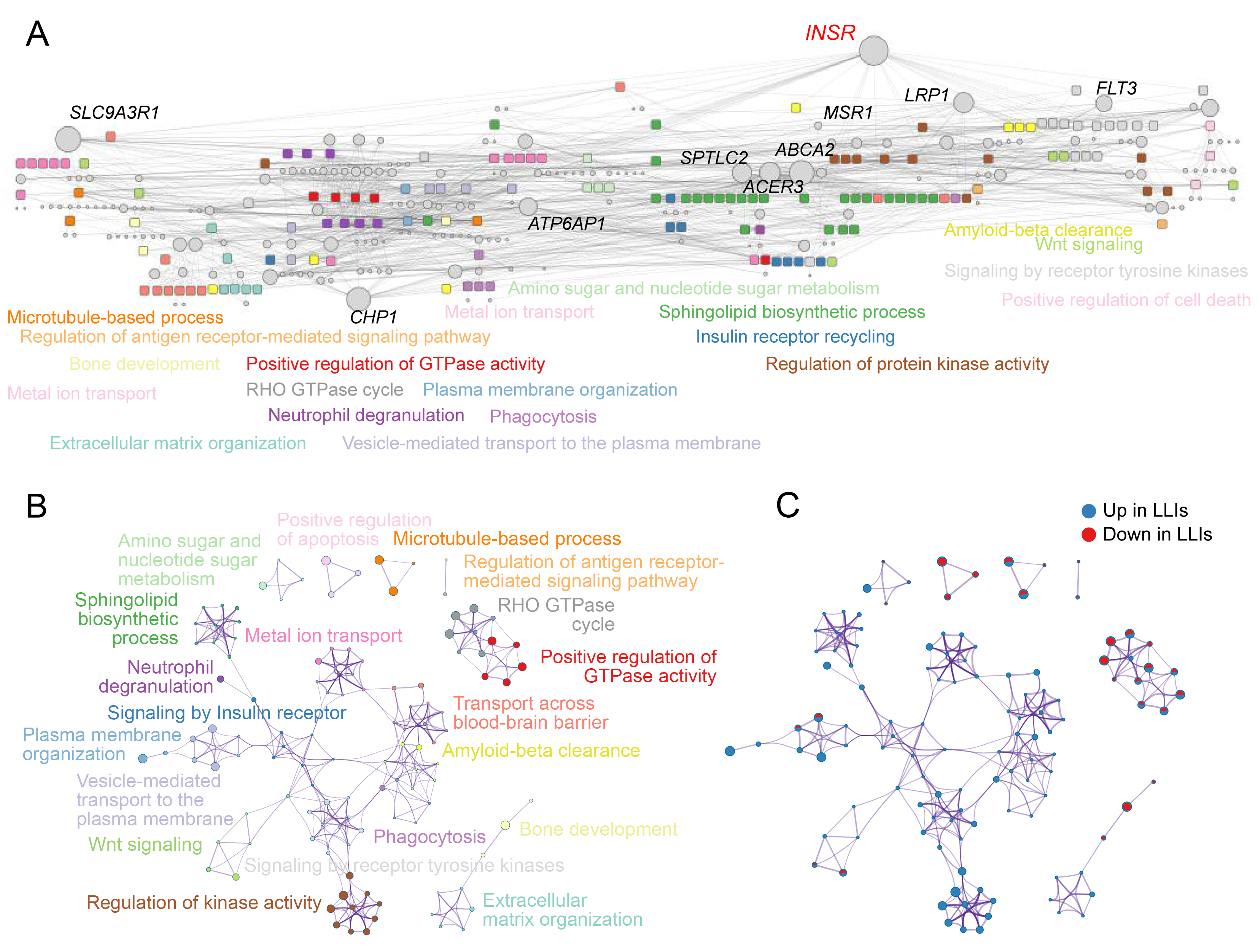

Supplement: Supplementary file 2 — Figure S2. [file ACEL-22-e13810-s012.tif]

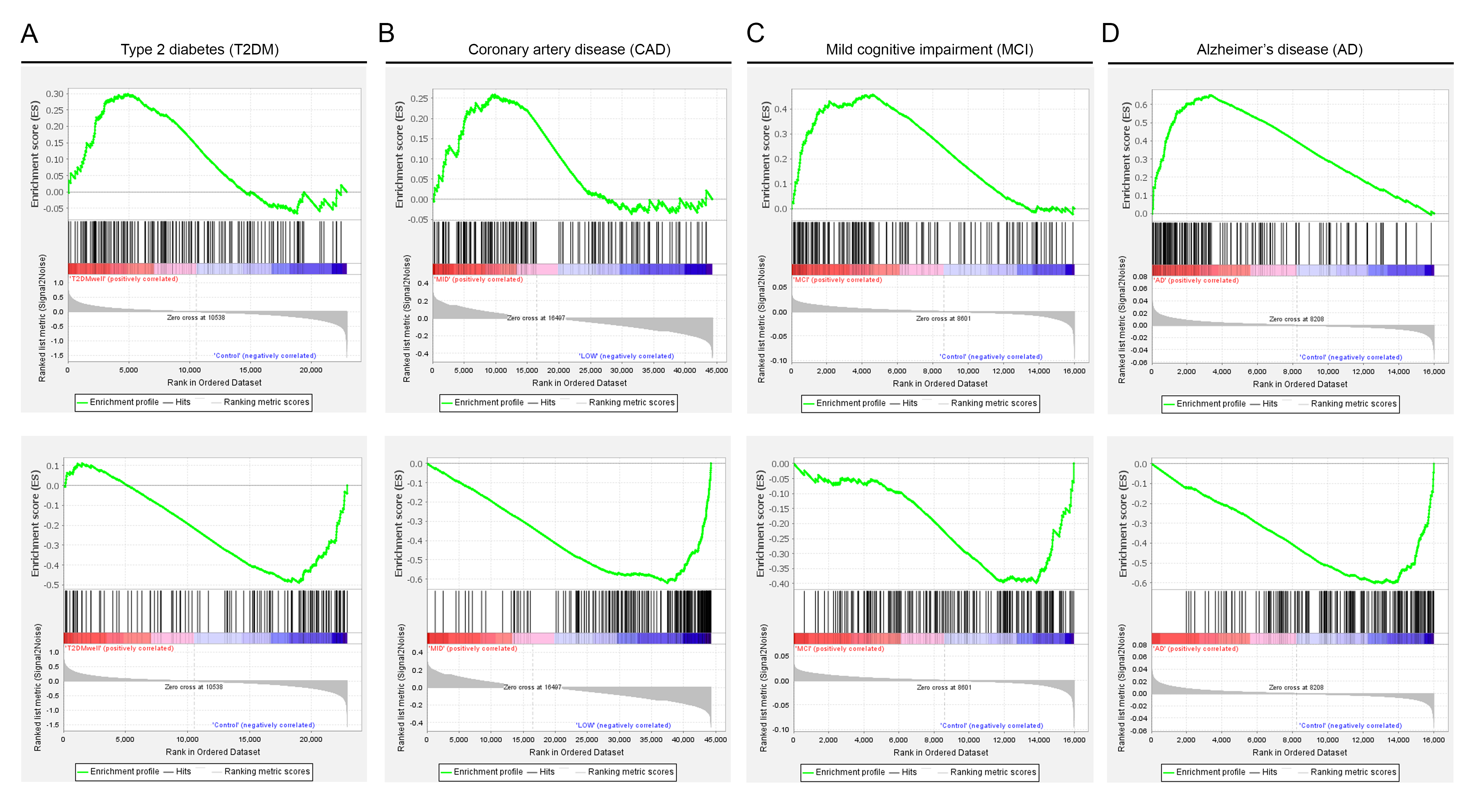

Supplement: Supplementary file 3 — Figure S3. [file ACEL-22-e13810-s019.tif]

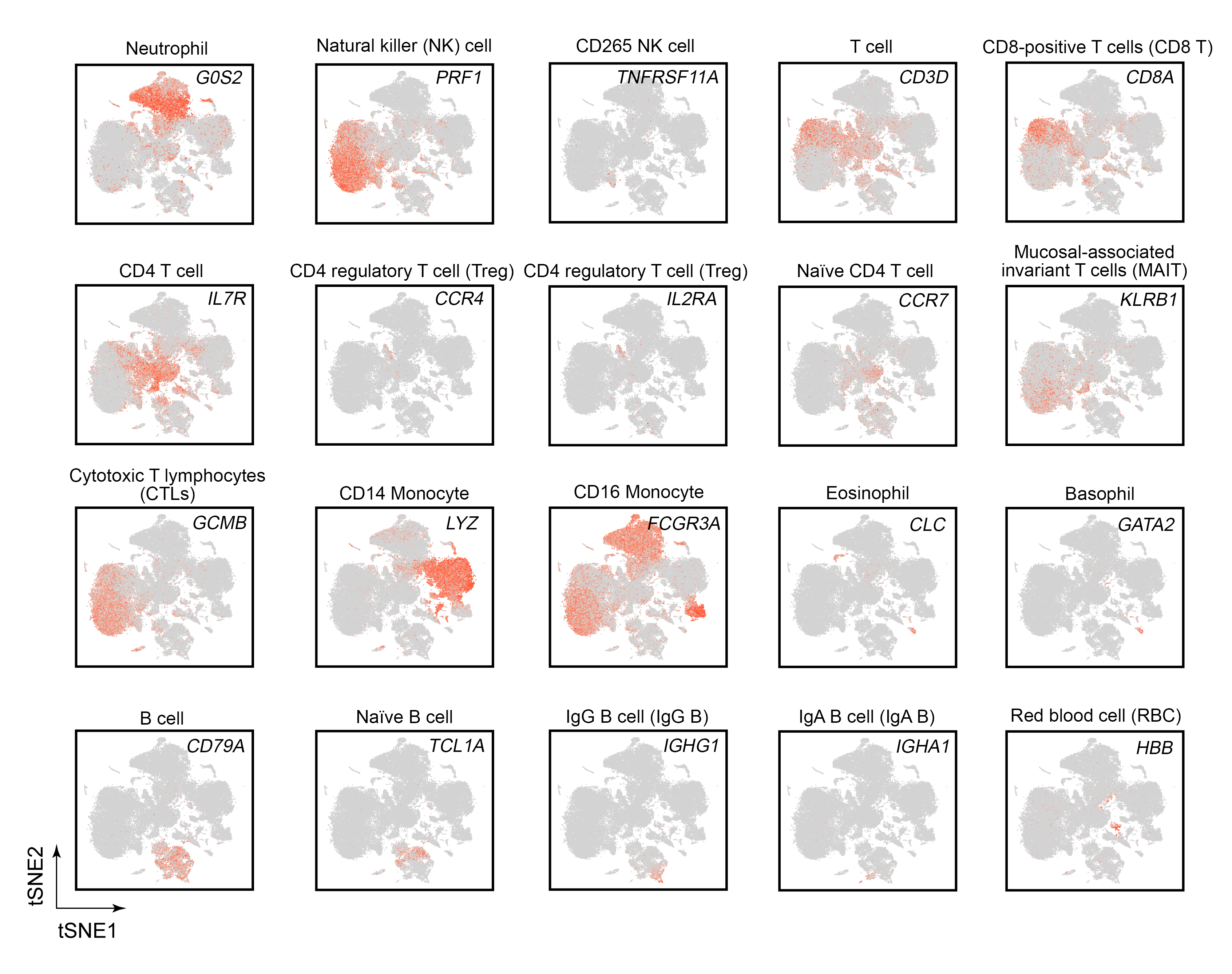

Supplement: Supplementary file 4 — Figure S4. [file ACEL-22-e13810-s014.tif]

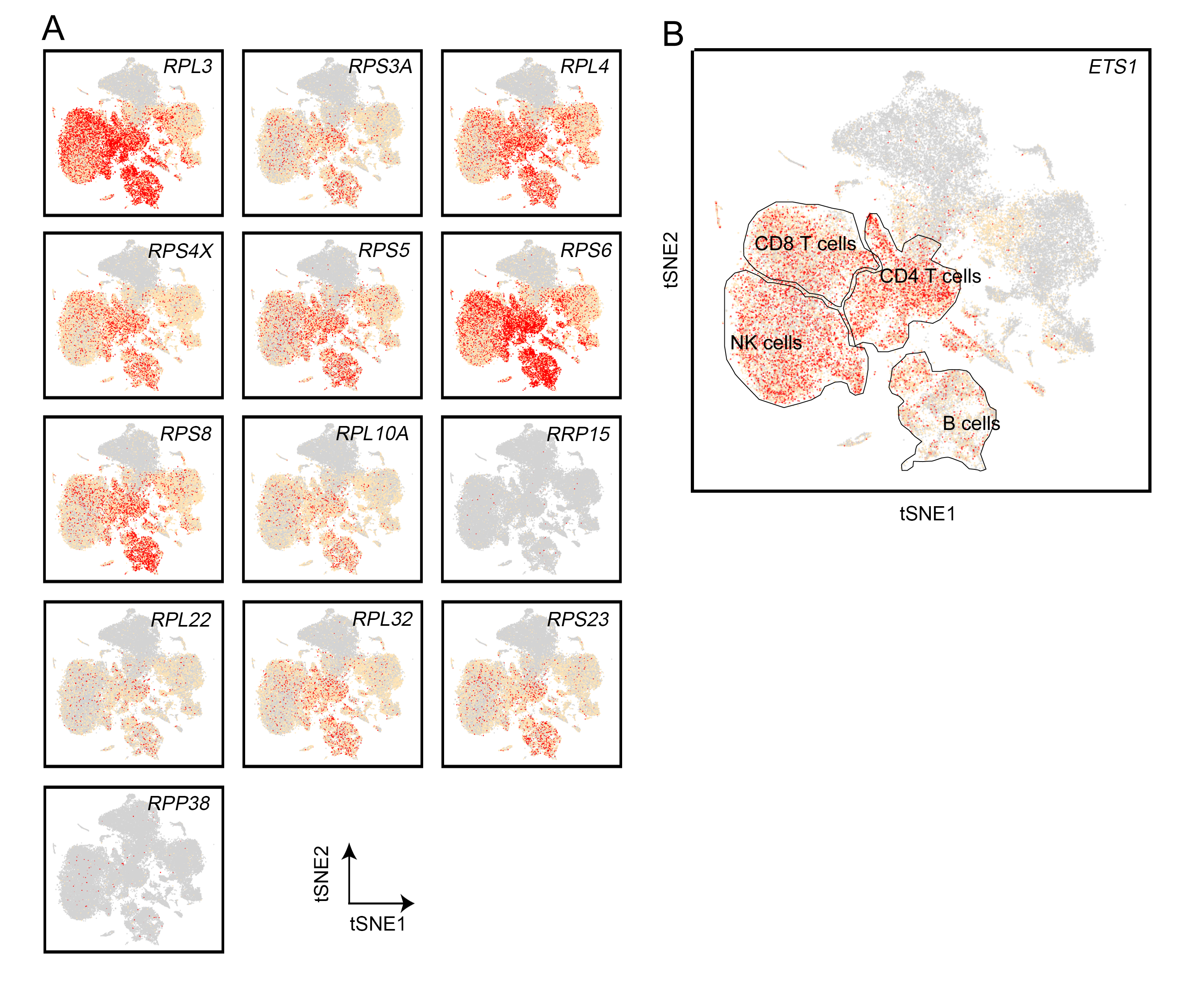

Supplement: Supplementary file 5 — Figure S5. [file ACEL-22-e13810-s020.tif]

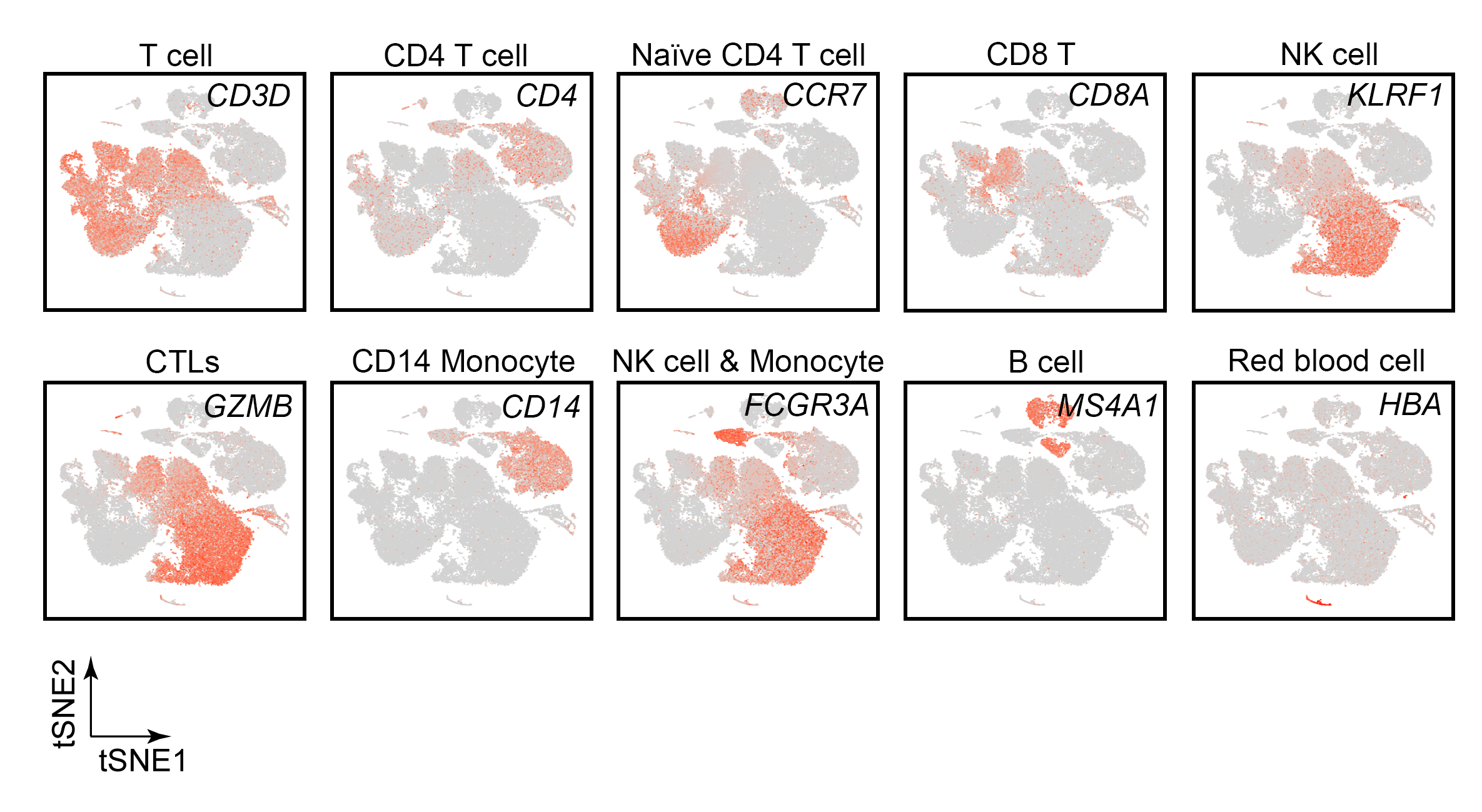

Supplement: Supplementary file 6 — Figure S6. [file ACEL-22-e13810-s007.tif]

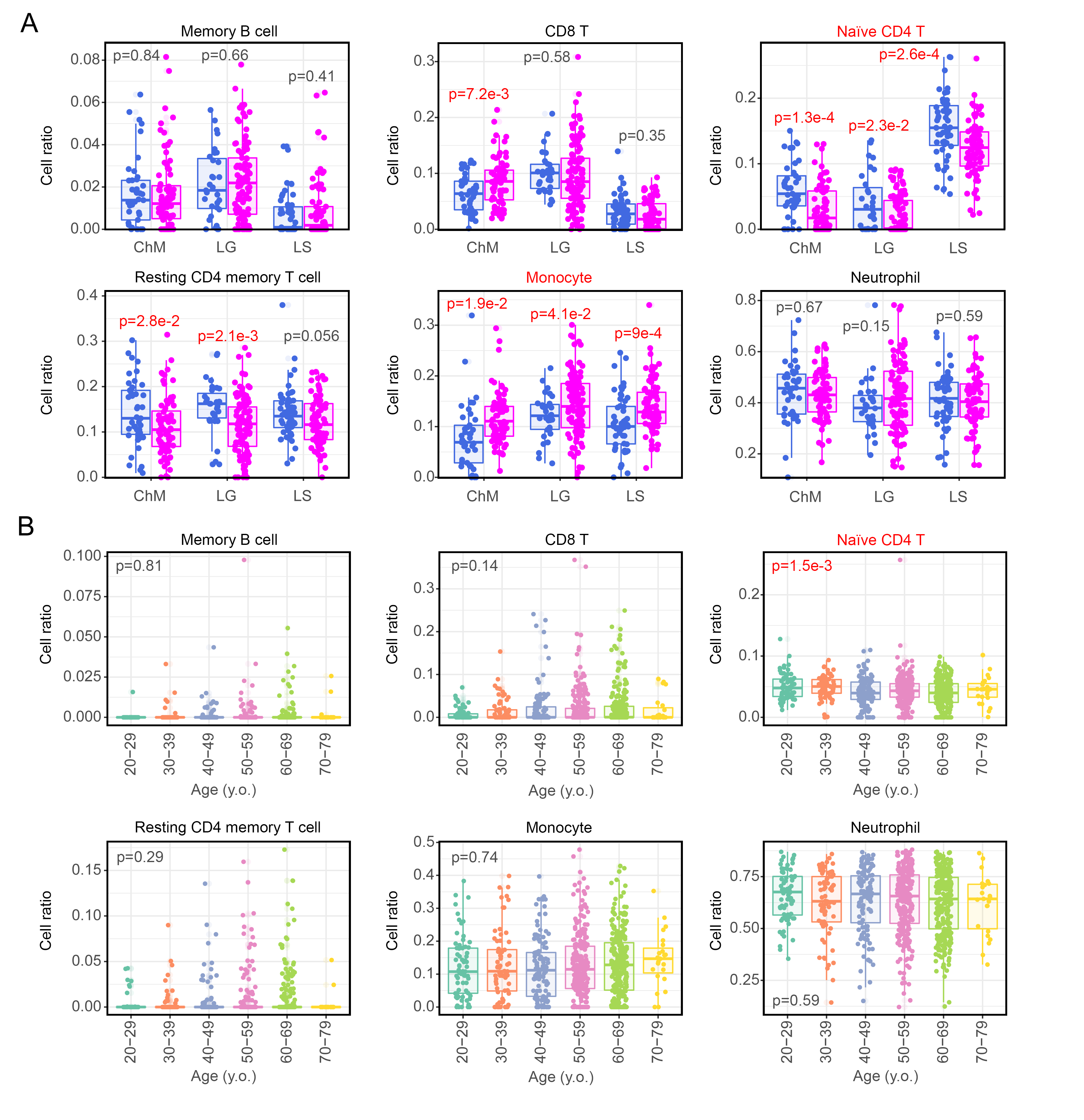

Supplement: Supplementary file 7 — Figure S7. [file ACEL-22-e13810-s018.tif]

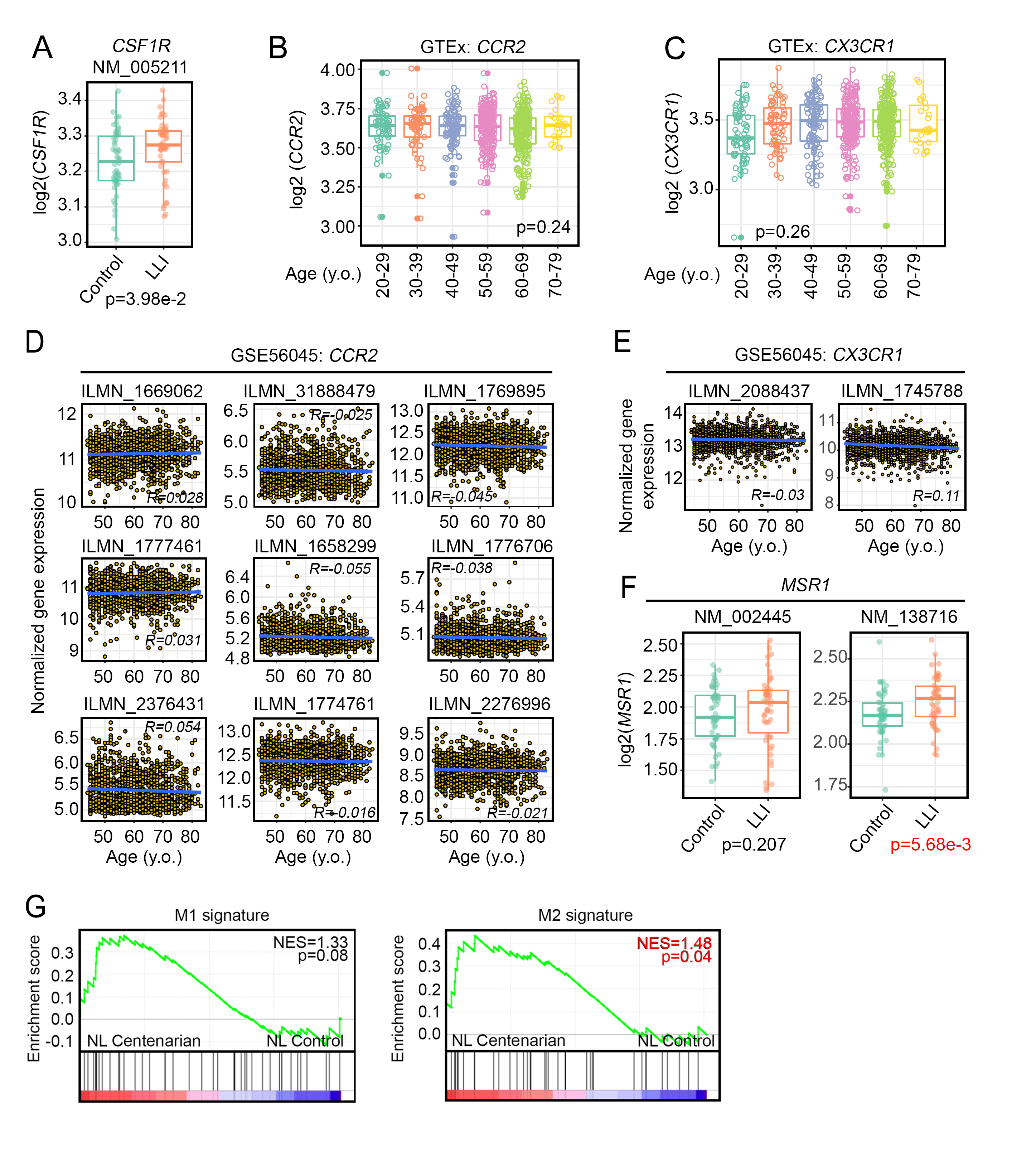

Supplement: Supplementary file 8 — Figure S8. [file ACEL-22-e13810-s024.tif]

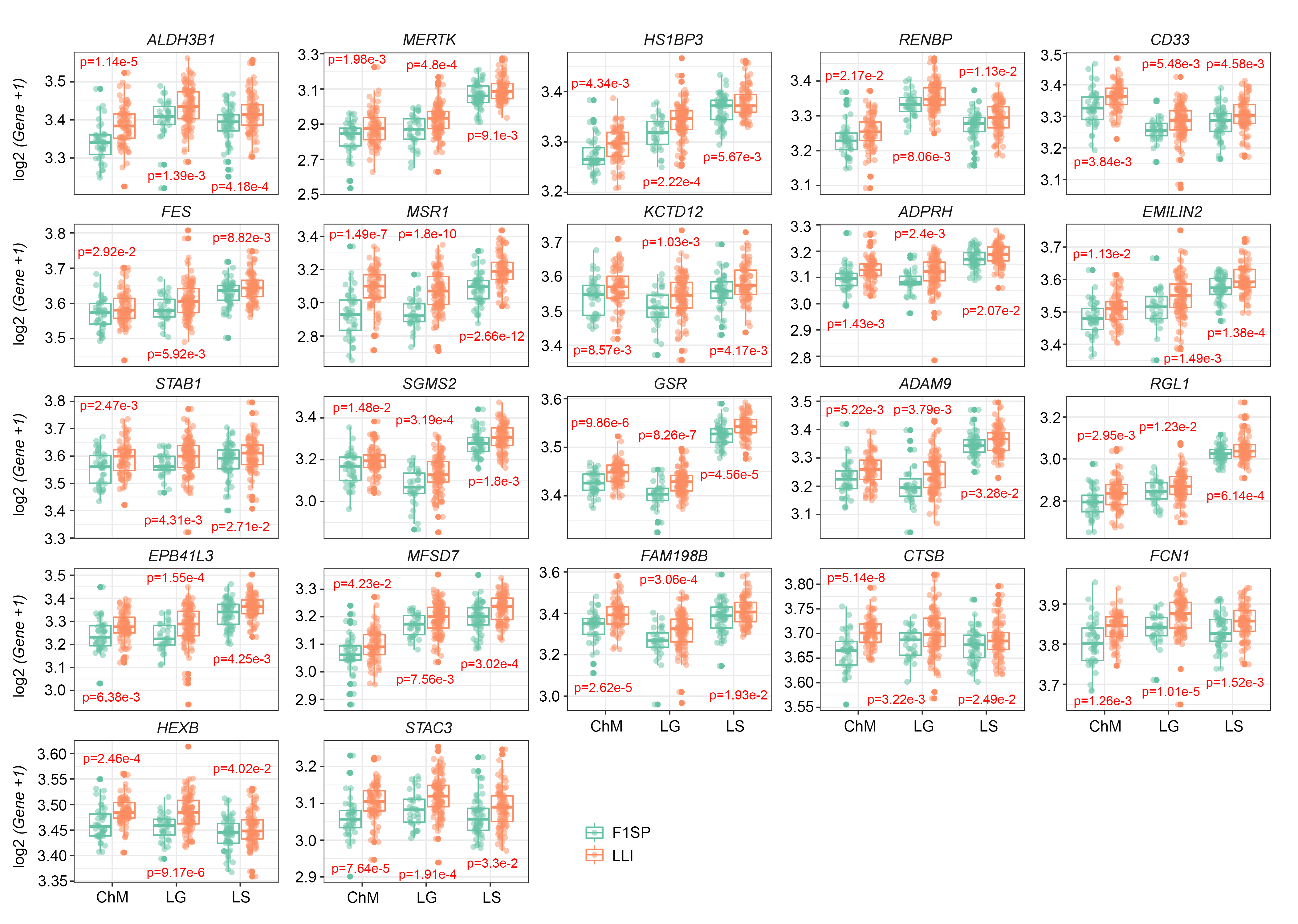

Supplement: Supplementary file 9 — Figure S9. [file ACEL-22-e13810-s010.tif]

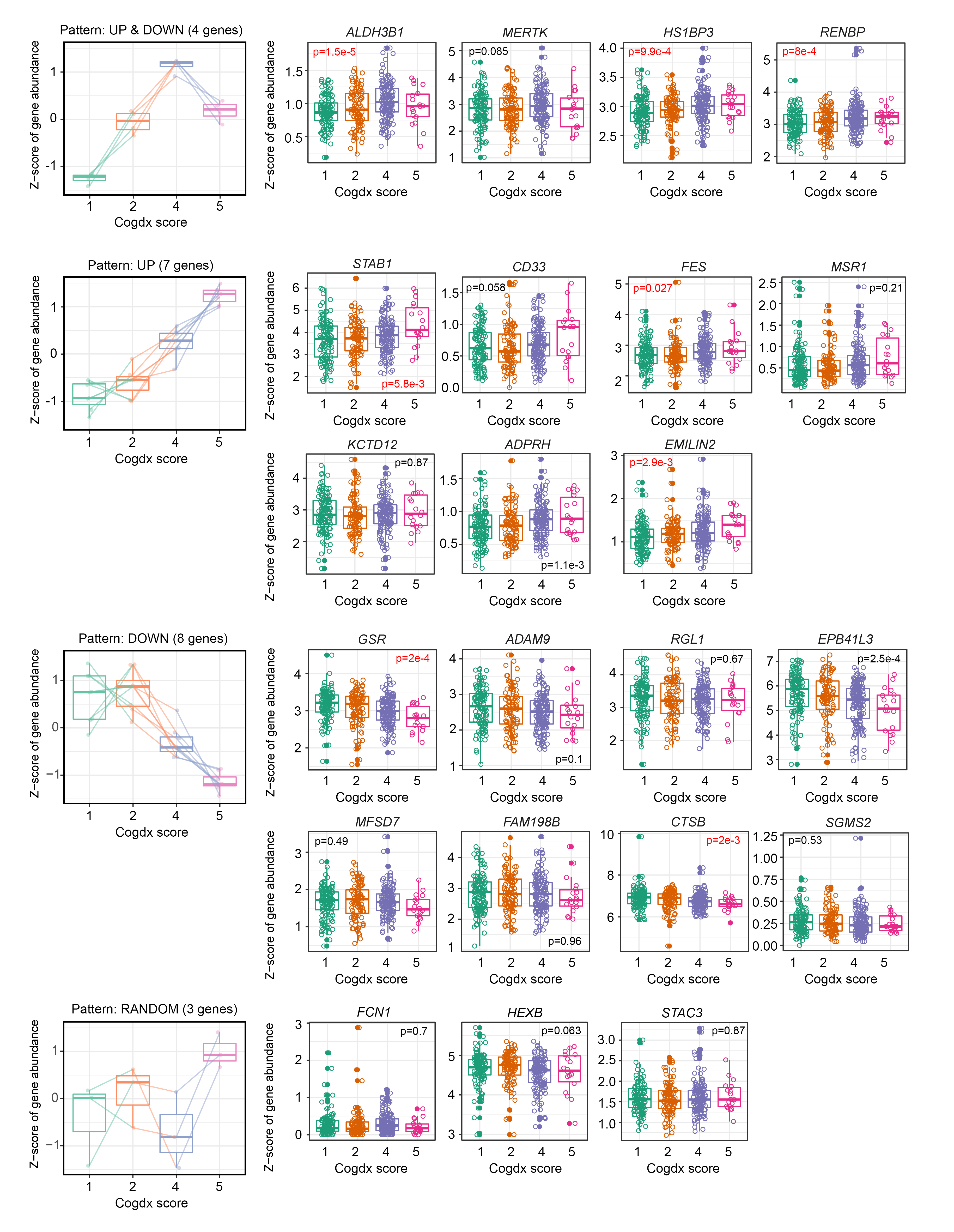

Supplement: Supplementary file 10 — Figure S10. [file ACEL-22-e13810-s017.tif]

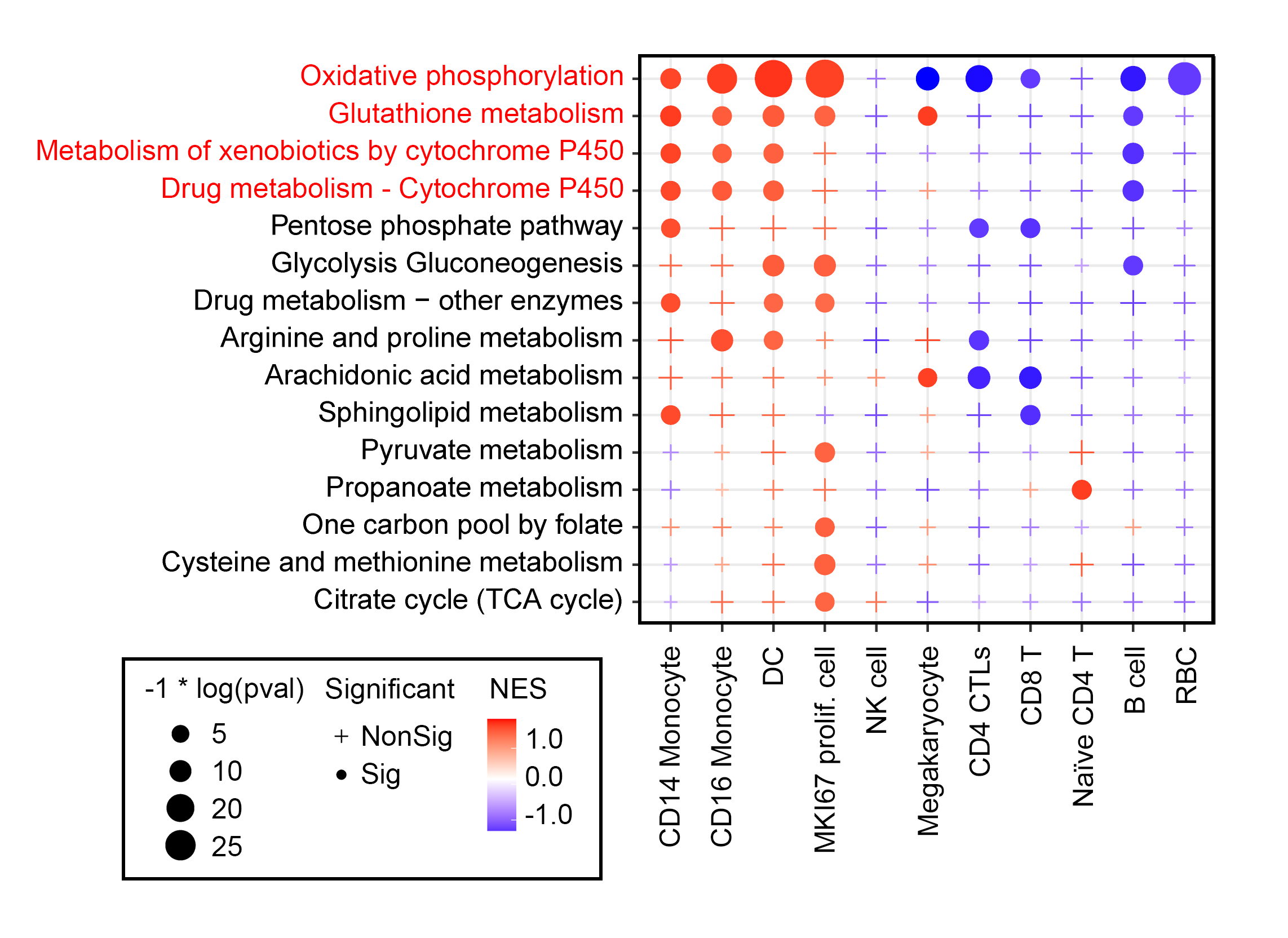

Supplement: Supplementary file 11 — Figure S11. [file ACEL-22-e13810-s021.tif]

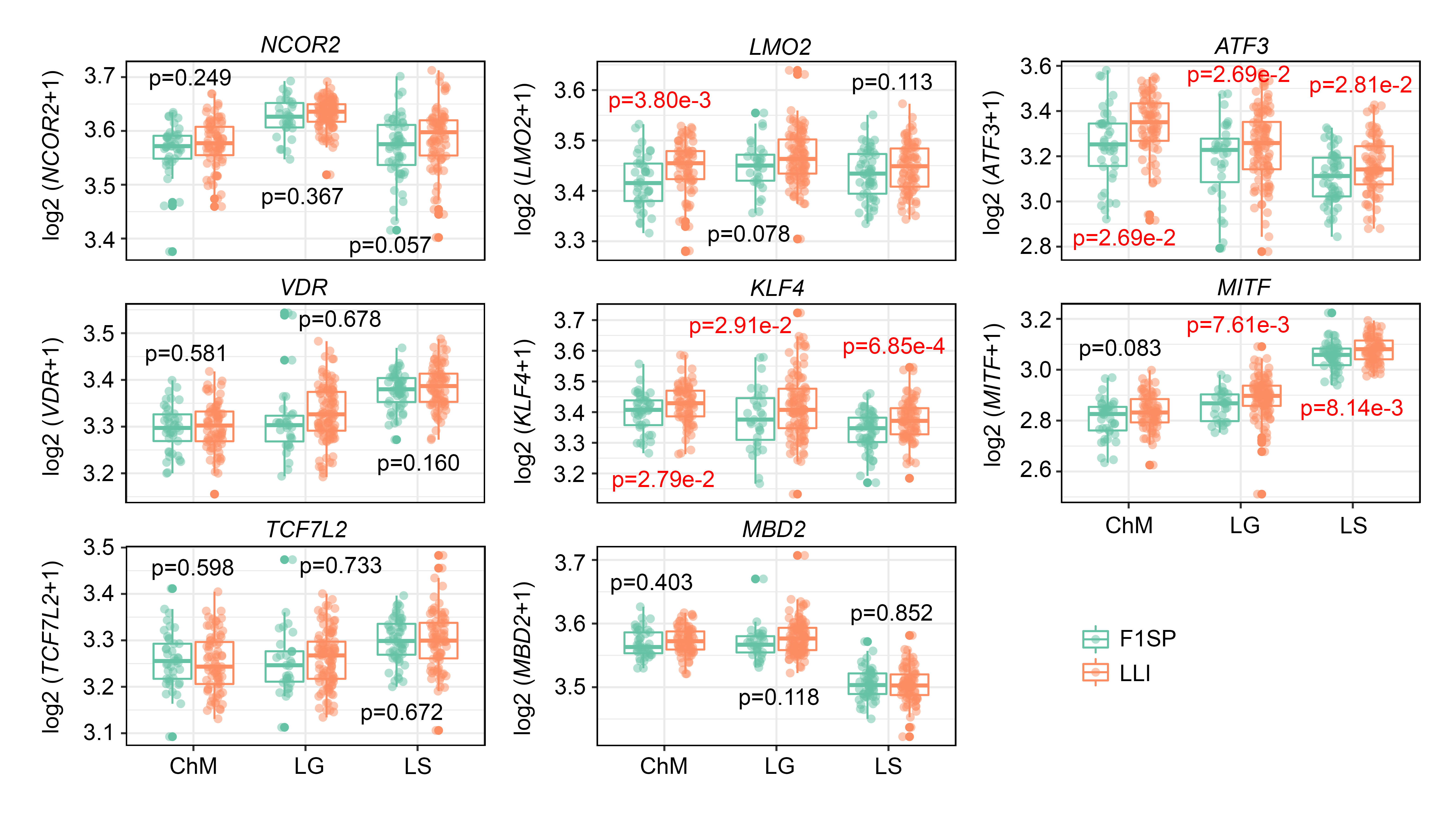

Supplement: Supplementary file 12 — Figure S12. [file ACEL-22-e13810-s022.tif]
